# Nuclear porcupine mediates XRCC6/Ku70 *S-*palmitoylation in the DNA damage response

**DOI:** 10.1186/s40164-024-00572-w

**Published:** 2024-11-04

**Authors:** Yang Chen, Mingming Xiao, Yaqi Mo, Jinlu Ma, Yamei Han, Qing Li, Qinghua Zeng, Rebecca J. Boohaker, Joshua Fried, Yonghe Li, Han Wang, Bo Xu

**Affiliations:** 1 Department of Biochemistry and Molecular Biology, The Key Laboratory of Breast Cancer Prevention and Therapy, Tianjin’s Clinical Research Center for Cancer, Ministry of Education, National Clinical Research Center for Cancer, Tianjin Medical University Cancer Institute and Hospital, Tianjin, 300060 China; 2 Chongqing Key Laboratory of Intelligent Oncology for Breast Cancer, Chongqing University Cancer Hospital and Chongqing University School of Medicine, Chongqing, 400030 China; 3 Department of Radiation Oncology, Tianjin Medical University Cancer Institute and Hospital, National Clinical Research Center for Cancer, Tianjin, 300060 China; 4 Department of Radiation Oncology, the First Affiliated Hospital, Xi’an Jiaotong University, Xi’an, 710061 China; 5 Department of Oncology, Southern Research Institute, Birmingham, AL 35205 USA; 6 Cell Biology Program, University of Alabama at Birmingham, Birmingham, AL 35205 USA; 7 Department of Pancreatic Surgery, Fudan University Shanghai Cancer Center, Shanghai, 200032 China

**Keywords:** DNA damage response, Porcupine, Palmitoylation, Ku70, Nonhomologous end joining repair

## Abstract

**Background:**

The activation of the DNA damage response (DDR) heavily relies on post-translational modifications (PTMs) of proteins, which play a crucial role in the prevention of genetic instability and tumorigenesis. Among these PTMs, palmitoylation is a highly conserved process that is dysregulated in numerous cancer types. However, its direct involvement in the DDR and the underlying mechanisms remain unclear.

**Methods:**

CRISPR-Cas9 technology was used to generate the PORCN KO and PORCN NLS KO cell lines. The effects of PORCN NLS in the DDR were verified by colony formation assays, MTT assays, the DR/EJ5 homologous recombination/non-homologous end-joining reporter system, xenograft tumor growth and immunofluorescence. Mechanisms were explored by mass spectrometry, acyl-biotin exchange (ABE) palmitoylation assay, Click-iT assay, cell subcellular fractionation assay, Western blot analysis, and in vivo and in vitro co-immunoprecipitation.

**Results:**

In this study, we introduce evidence that Porcupine (PORCN) is an integral component of and plays a critical role in the DDR. PORCN deficiency hampers nonhomologous end joining (NHEJ) and highly sensitizes cells to ionizing radiation (IR) both in vitro and in vivo. We also provide evidence that PORCN possesses a nuclear fraction (nPORCN) with *S-*acyltransferase activity, unlike its membrane-bound *O*-acyltransferase in the endoplasmic reticulum. Furthermore, we show that nPORCN is necessary for the successful activation of NHEJ. Using mass spectrometry, we reveal the existence of an nPORCN complex and show that nPORCN mediates the *S*-palmitoylation of XRCC6/Ku70 at five specific cysteine sites in response to IR. Mutation of these sites causes a substantial increase in radiosensitivity and delays NHEJ. Additionally, we present evidence that nPORCN-dependent Ku70 palmitoylation is required for DNA-PKcs/Ku70/Ku80 complex formation.

**Conclusion:**

Our findings underscore the crucial role of nPORCN-dependent Ku70 S-palmitoylation in the DDR.

**Supplementary Information:**

The online version contains supplementary material available at 10.1186/s40164-024-00572-w.

## Background

Genomic instability is regarded as a hallmark of cancer [1]. One major mechanism that guards against genome instability is the DNA damage response (DDR). In response to endogenous and/or exogenous (such as ionizing radiation [IR] or ultraviolet radiation [UV]) stress, the DDR initiates cell cycle checkpoints, DNA repair and programmed cell death in an organized way [2]. Exposure to IR causes DNA double-strand breaks (DSBs), which are the most deleterious form of DNA damage and affect both strands of the DNA duplex. DSBs are repaired by two specific mechanisms: both ends can be simply rejoined with little or no further processing, a process referred to as nonhomologous end joining (NHEJ), or they can be repaired by homologous recombination (HR). NHEJ utilizes proteins to repair DNA ends in a flexible manner. In contrast, HR provides high-fidelity, template-dependent repair. Upstream elements critical for the activation of the DDR in response to DSBs include ATM/ATR kinases and the DNA-dependent protein kinase catalytic subunit (DNA-PKcs) [3]. A key component of the DNA-PK complex is the Ku heterodimer, composed of the Ku70 (XRCC6) and Ku80 (XRCC5) subunits [4]. These are the initial DNA end-binding components of the NHEJ complex. The proteins involved in HR include Rad51, BRCA1, BRCA2, and BARD1 [5–8].

The activation of the DDR involves protein PTMs, such as phosphorylation, acetylation, methylation, palmitoylation, ubiquitination and sumoylation [9–13]. Among them, palmitoylation is the lipid modification of a protein with the 16-carbon fatty acid palmitate, and it is highly conserved in eukaryotes [14]. Palmitoylation is dynamic, and cycling between palmitoylation and depalmitoylation states [15]. There are three major types of palmitoylation: *S-*, *N*- and *O-*palmitoylation [16]. *S-*palmitoylation is the most common one. Approximately 90% of palmitoylation proteins are catalysed by DHHC palmitoyl acyltransferases (PATs) [16]. *N*-palmitoylation is catalysed by Hedgehog acyltransferase (HHAT), which attaches palmitate to cysteines at the N-terminus by a stable amide bond [14, 17]. The *O*-palmitoylation of proteins is catalysed by Porcupine (PORCN) and ghrelin acyltransferase (GOAT), which exhibit membrane-bound *O*-acyltransferase (MBOAT) activity and attach palmitate to an internal serine residue [18]. Dysregulated protein palmitoylation is associated with human diseases, such as neurological disorders, non-alcoholic steatohepatitis, and cancers [16, 19, 20]. In cancer, increasing evidence has shown that palmitoylation of many oncoproteins (e.g., EGFR, RAS, PD1/PD-L1) and tumour suppressors (e.g., SCRIB, melanocortin 1 receptor) alters signal transduction in tumour cells, ultimately affecting the development of tumours and determining sensitivity to cancer treatment [21–25].

PORCN has diverse biological functions, such as cell differentiation, proliferation, migration, and apoptosis. Known as an endoplasmic reticulum protein, PORCN palmitoylates Wnt proteins, promoting their secretion to the plasma membrane and activation of cellular responses [26]. The Wnt/β-catenin pathway is fundamental for embryonic development, adult stem cell maintenance and oncogenesis [27–30]. In cancer cells, palmitoylation is essential for the secretion and binding of all mammalian Wnts to their FZD receptors [26]. Inhibition of PORCN eliminates the palmitoylation of Wnt ligands and prevents the transport of Wnt to the extracellular membrane, thus preventing the production of β-catenin and aberrant cell growth [31].

Clinically, PORCN inhibitors have been shown to suppress growth and progression in multiple cancers. Among them, LGK974, ETC-159, CGX1321 and RXC004 have entered phase I clinical trials for patients with selected solid malignancies [18]. It has been reported that ETC-159 synergizes with the PARP inhibitor olaparib in Wnt-addicted cancers by affecting HR and the Fanconi anemia (FA) pathway [32]. In addition, the intestines of PORCN-deficient mice are more sensitive to ionizing radiation (IR) [33]. These results indicate that PORCN might be involved in the DDR, although the mechanism remains to be elucidated.

In this study, we demonstrate that PORCN is directly involved in NHEJ and radiosensitivity. PORCN is an endoplasmic reticulum protein; however, we show that a fraction of PORCN exists in the nucleus (nPORCN) and has *S*-acetyltransferase activity. Interestingly, the main proteins that interact with nPORCN are the DNA-PKcs/Ku70/Ku80 complex, and PORCN palmitoylates Ku70 at five cysteine sites. Our findings indicate that nPORCN-mediated Ku70 *S-*palmitoylation is required for DNA-PK complex formation and NHEJ. This study provides the first evidence that protein palmitoylation directly participates in NHEJ to prevent genetic instability.

## Methods

### Cell lines and culture

HT1080, HeLa, HT29, MCF-7, MDA-MB-231 and HEK293T cells were obtained from the American Type Culture Collection (Manassas, VA, USA) and cultured according to the manufacturer’s instructions. DR-U2OS and EJ5-U2OS cells were kindly provided by Dr. Lei Shi (Tianjin Medical University). PORCN-null and control HT1080 cells were kindly provided by Dr. David M. Virshup (Duke-National University of Singapore). These cells were cultured in DMEM supplemented with 10% foetal bovine serum (FBS), 100 U/ml penicillin and 100 U/ml streptomycin and were maintained at 37 °C in a humidified atmosphere with 5% CO_2_.

### Plasmid construction, lentivirus production, and transfection

The target sequence of PORCN used to construct lentiviral shRNAs was 5′-ATCTTCTACCGTCTCATAGT-3′ (shPORCN). For rescue experiments, PORCN-WT and a PORCN-ΔNLS construct (mutations underlined: AT*A*TT*T*TA*T*CG*C*CT*A*AT*C*GT) resistant to the shRNA used (ATCTTCTACCGTCTCATAGT) were synthesized by Genewiz from Azenta Life Sciences. The target sequence of Ku70 used to construct lentiviral shRNAs was 5′-GATGAGTCATAAGAGGATCAT-3′ (shKu70). For rescue experiments, a Ku70-WT construct (mutations underlined: *A*ATG*TCC*CA*C*AA*AC*G*C*AT*A*AT) resistant to the shRNA used (GATGAGTCATAAGAGGATCAT) was cloned and inserted into a pLenti-Hygro vector. The QuikChange II XL Site-Directed Mutagenesis Kit (Stratagene, La Jolla, CA, USA) was used to generate the Ku70-C66S, C150S, C389S, C398S, C585S and 5 C/S plasmids. To generate stable PORCN- or Ku70-knockdown cell lines, HEK293T cells were used to produce retrovirus by cotransfection with PORCN shRNA or Ku70 shRNA packaged in the pCMV-VSVG, pRsv-REV and pMDlg-pRRE plasmids using Lipofectamine 3000. Forty-eight hours after transfection, retroviruses were harvested. To generate stable cell lines, HT1080 cells were infected with retroviruses in the presence of polybrene (8 µg/mL). Cells were then selected with puromycin (0.7 µg/mL) or hygromycin B (400 µg/mL).

### Generation of PORCN knockout (PORCN-KO) and NLS knockout (NLS-KO) cell lines

For PORCN deletion, we designed two sgRNAs targeting a common sequence in all PORCN transcripts to delete a large fragment of PORCN-CDS. The sgRNA sequences were as follows: gRNA-A3: CTGGGTAGAGGGCGTAGCCAGGG and gRNA-A4: CAGGGAGCGCAGATATATGGGGG. By analysing the PORCN genome, we found that the NLS sequence occupies the entire exon 6 of PORCN. Thus, for PORCN NLS deletion, we deleted exon 6 of PORCN in HT1080 cells using CRISPR/Cas9 gene editing. Two sgRNAs were designed to delete the fragment. The sgRNA sequences were as follows: gRNA-A1: CTCAGAACTGGGGAAACCGCGGG and gRNA-A2: GCTGGCTGGCTGACGAAGCTTGG. The positive monoclony was obtained after PCR and sequencing verification.

### Irradiation

For ionizing radiation, a 6 MV X-ray linear accelerator (600CD, Varian, USA) was used at a dose rate of 2.3 Gy/min for the in vitro studies.

### Colony formation assay

Different numbers of HT-1080 cells were cultured in 6-well plates and treated with various doses of IR. The cells were then cultured for 10–14 days. The colonies were then fixed with 4% paraformaldehyde and stained with 0.5% crystal violet. Colonies with more than 50 cells were counted as surviving colonies. Plating efficiencies and surviving fractions were calculated.

### 3-(4,5-Dimethylthiazol-2-yl)-2,5-diphenyltetrazolium bromide (MTT) staining

MTT (Acros Organics, NJ, USA) was added to the cell culture medium at a 1:10 ratio. The cells were then placed back in the incubator until the MTT was metabolized. PBS was then added to the medium at a 1:10 ratio, and the plates were covered and kept overnight at 4 °C. Absorbance was read at 570 nm by a Synergy 4 plate reader (Biotek, Winooski, VT, USA).

### Immunofluorescence microscopy

To detect γ-H2AX foci, exponentially growing cells on coverslips were treated with 0–5 Gy IR. At the indicated timepoint after IR, the cells were fixed in 4% paraformaldehyde at 4 °C, permeabilized with 0.2% Triton X-100 and blocked with 5% BSA. The cells were then incubated with a rabbit anti-γ-H2AX antibody (#9718, Cell Signaling Technology) overnight at 4 °C. The cells were then incubated with a fluorescently labelled goat anti-rabbit secondary antibody (35560, Thermo Scientific) for 1 h at room temperature. DAPI staining (blue) was used to visualize the DNA among the bound antibodies detected by confocal microscopy (Olympus BX61, Japan). At least 100 cells from each experiment were randomly selected to count the number of γ-H2AX foci in each nucleus.

### HR and NHEJ reporter assays

DR-U2OS or EJ5-U2OS cells were harvested 48 h after transfection with I-SceI and other indicated plasmids. They were resuspended in 0.5 ml of PBS for fluorescence-activated cell sorting analysis. The ratio of GFP-positive cells was determined to assess repair efficiency.

### Comet assay

Cells were mixed gently with low-temperature-melting agarose at a volume ratio of 1:50 (v/v) and spread onto glass slides. The slides were then incubated in lysis buffer at 4 °C for 30 min before they were electrophoresed in running buffer at 1.0 V/cm for 15 min and stained with Subgreen in TE buffer (1:10000).

### Giemsa staining

Cells were fixed with 70% ethanol and stained with Giemsa stain solution (G4640, Solarbio, Beijing, China).

### G2/M checkpoint assay

To test the activation of the G2/M checkpoint, cells were harvested and fixed in 70% ethanol, permeabilized with 0.1% Triton X-100 (Sigma) on ice for 10 min and blocked in 1% bovine serum albumin for 30 min. The fixed cells were then incubated with an anti-phospho-histone-H3-Ser10 antibody (#53348; Cell Signaling Technology) for 1 h and a fluorescein isothiocyanate–conjugated secondary antibody for 30 min in the dark. In the final step, the cells were stained with 50 µg/ml propidium iodide and analysed using a fluorescence-activated cell sorting Calibur flow cytometer (Becton, Dickinson and Company) with CellQuest software (Becton, Dickinson and Company).

### Cell subcellular fractionation assay

Different cellular compartments of HT1080 cells were isolated using a commercial Subcellular Protein Fractionation Kit (78840, Thermo Scientific). Briefly, gently mix the cell pellet and CEB together, then incubate the tube at 4 °C for 10 min. Centrifuge for five minutes at 500 ×g. Quickly move the cytoplasmic extract, or supernatant, to a sterile, ice-filled tube that has been previously refrigerated. To the pellet, add ice-cold MEB with protease inhibitors. Using the highest setting, vortex the tube for 5 s. Gently stir the tube while it is incubating at 4 °C for 10 min. Centrifuge for five minutes at 3000 ×g. Place the membrane extract, or supernatant, in a clean, previously refrigerated tube and set it on ice. To the pellet, add ice-cold NEB that has protease inhibitors in it. 15 s at the maximum vortex setting. Gently mix the tube and incubate it for 30 min at 4 °C. Centrifuge for five minutes at 5000×g. Place the nuclear extract fraction (supernatant) in a clean, ice-filled tube that has been previously refrigerated. To make the chromatin-bound extraction buffer, mix 3µL of 300-unit Micrococcal Nuclease and 5µL of 100-milligram CaCl_2_ with 100µL of room-temperature NEB. To the pellet, add room temperature NEB that contains micrococcal nuclease, CaCl_2_, and protease inhibitors. 15 s at the maximum vortex setting. Incubate for fifteen minutes at room temperature or five minutes in a water bath at 37 °C. Following the incubation period, centrifuge the tube at 16,000 ×g for 5 min and vortex on the highest setting for 15 s. Place the chromatin-bound nuclear extract (supernatant) fraction in a clean, ice-filled tube that has been previously refrigerated. To the pellet, add room temperature PEB that contains protease inhibitors. 15 s at the maximum vortex setting. For ten minutes, incubate at room temperature. For five minutes, centrifuge the tube at 16,000 ×g. In a fresh tube, transfer the supernatant. The isolated fractions were processed for Western blot analysis.

### Immunoprecipitation, LC-MS/MS and western blot analysis

Cells were collected, washed with cold PBS, and lysed in RIPA buffer (50mM Tris, 150mM NaCl, 1% Triton X-100, 1% sodium deoxycholate, pH 7.4). Cell lysates were then immunoprecipitated with the indicated antibodies and protein A/G Sepharose overnight and eluted with Laemmli buffer after washing. The interacting proteins of PORCN were analysed and visualized on SDS-PAGE gel by silver staining following the manufacturer’s protocol (24600, Thermo Scientific). Each gel lane was divided into fractions and digested with trypsin. The tryptic peptides were analysed by liquid chromatography-MS/MS using an LTQ Orbitrap Velos Pro ion-trap mass spectrometer. For Western blot, protein lysates were separated using SDS-PAGE (8–12%) and transferred to polyvinylidene difluoride (PVDF) membranes. The membranes were blocked in 5% non-fat dry milk in Tris-buffered saline and Tween 20 (TBST) for 2 h at room temperature and then incubated overnight at 4 °C with primary antibodies against PORCN (ab105543, Abcam), DNA-PKcs (ab70250, Abcam), pDNA-PKcs(S2056)(ab124918, Abcam), Ku70 (ab202022, Abcam), Ku80 (ab80592, Abcam), α-Tubulin (ab18251, Abcam), Histone H1 (ab203337, Abcam), Rad50 (#3427, Cell Signaling Technology), γ-H2AX (#9718, Cell Signaling Technology), Flag (F1804, Sigma), GFP (50430-2-AP, Proteintech), Streptavidin-HRP (21130, Pierce), LRP6 (#3395, Cell Signaling Technology), p-LRP6(S1490) (#2568, Cell Signaling Technology), ATM (#2873, Cell Signaling Technology), pATM(S1981) (#5883, Cell Signaling Technology), Total β-Catenin(#8814, Cell Signaling Technology), Free β-Catenin(#9561, Cell Signaling Technology), GAPDH (#5174, Cell Signaling Technology) and β-actin (#3700, Cell Signaling Technology). The next day, the membranes were incubated with horseradish peroxidase-conjugated goat anti-rabbit/mouse secondary antibodies (#7074/#7076, Cell Signaling Technology). After 1 h at RT, the band intensities were quantified and analysed with Quantity One 1 image analysis software (Bio-Rad).

### In vitro coimmunoprecipitation (co-IP) assay

In vitro co-IP assays were performed in PBS in a total volume of 200 µl using 10 µl of protein A/G beads combined with IgG or an anti-PORCN antibody. Each reaction contained 1 µg of GST-tagged Ku70 protein (H00002547-P01, Abnova) and 1 µg of recombinant PORCN protein (CSB-CF887958HU(A4), Cusabio). Binding reactions were incubated at 4 °C for 2 h with rotation. The beads were then washed extensively in PBS. The proteins were then eluted with SDS loading buffer and analysed by Western blot.

### Click-iT chemistry assay

Cells were incubated with 50 µM click-iT palmitic acid-azide (C10265, Thermo Scientific) at 37 °C in 5% CO_2_ for 8 h, washed three times with PBS and lysed with lysis buffer (1% sodium dodecyl sulphate in 50 mM Tris-HCl, pH 8.0). The protein samples were allowed to react with biotin-alkyne using the Click-iT Protein Reaction Buffer Kit (C10276, Thermo Scientific). The biotin alkyne-azide-palmitic protein complex was then pulled down by streptavidin, and the pellets were treated with 1 M hydroxylamine (HAM) in 1% Triton X-100 (pH 7.2) for 1 h at room temperature and then subjected to immunoblotting.

### Acyl-biotin exchange (ABE) palmitoylation assay

To irreversibly block unmodified thiol groups, cells were lysed in the presence of N-ethylmaleimide (NEM) in lysis buffer (100 mM NEM, 1% Triton X-100, 1x protease inhibitor cocktail, 1 mM phenylmethanesulfonylfluoride). The lysates were then immunoprecipitated with an anti-Ku70 antibody. G-Sepharose (IP05, Millipore)-coupled precipitates were then incubated with HAM buffer (1 h, RT, LB pH 7.2, 1 M HAM) for specific cleavage and unmasking of the palmitoylated cysteine thiol group. Thereafter, the beads were gently washed with lysis buffer (pH 6.2) and incubated in biotin-BMCC buffer (LB pH 6.2, 1 µM biotin-BMCC, 1 h, 4 °C) for selective labelling of the palmitoylated cysteines. The beads were finally resuspended in 90 µl of nonreducing SDS-PAGE sample buffer; then SDS-PAGE and Western blot with anti-streptavidin-HRP were performed.

### Three-dimensional structural model of Ku70

The amino acid sequence of human KU70 was obtained from the UniProt database (ID: P12956). However, since a complete crystal structure for KU70 was unavailable, we utilized ALPHAFOLD2 AI homology modelling software to create a three-dimensional structural model of Ku70. The validity of the model structure was then evaluated using tools such as PROCHECK, which revealed that the essential amino acids were present within reliable structural intervals (favourable and accessible region > 98%).

### The xenograft model

Animal procedures were approved by the Ethics Committee of the Tianjin Medical University Cancer Institute and Hospital. Female Nu/Nu mice (6 weeks old, 16–18 g) were used for xenograft experiments. The mice were randomized into six groups (6 or 8/group). Isogenic HT1080 cells and control (1 × 10^6^ in 0.2 ml normal saline) were injected subcutaneously into the right thigh of mice. When the average tumour volume reached 200 mm^3^, the xenografts were irradiated with 0–10 Gy with shielding of the surrounding areas. Tumour size was measured in two dimensions every 3 days for 33 days.

### Statistical analysis

The Statistical Product and Service Solutions 18.0 software package (IBM Corporation, Armonk, NY, USA) was used for statistical analyses. Two-way ANOVA or Student’s *t test* was used to calculate the significance of the differences between the control and treatment groups. *p < 0.05, **p < 0.01, and ***p < 0.001.

## Results

### PORCN is involved in the regulation of radiosensitivity and NHEJ

**Fig. 1:**
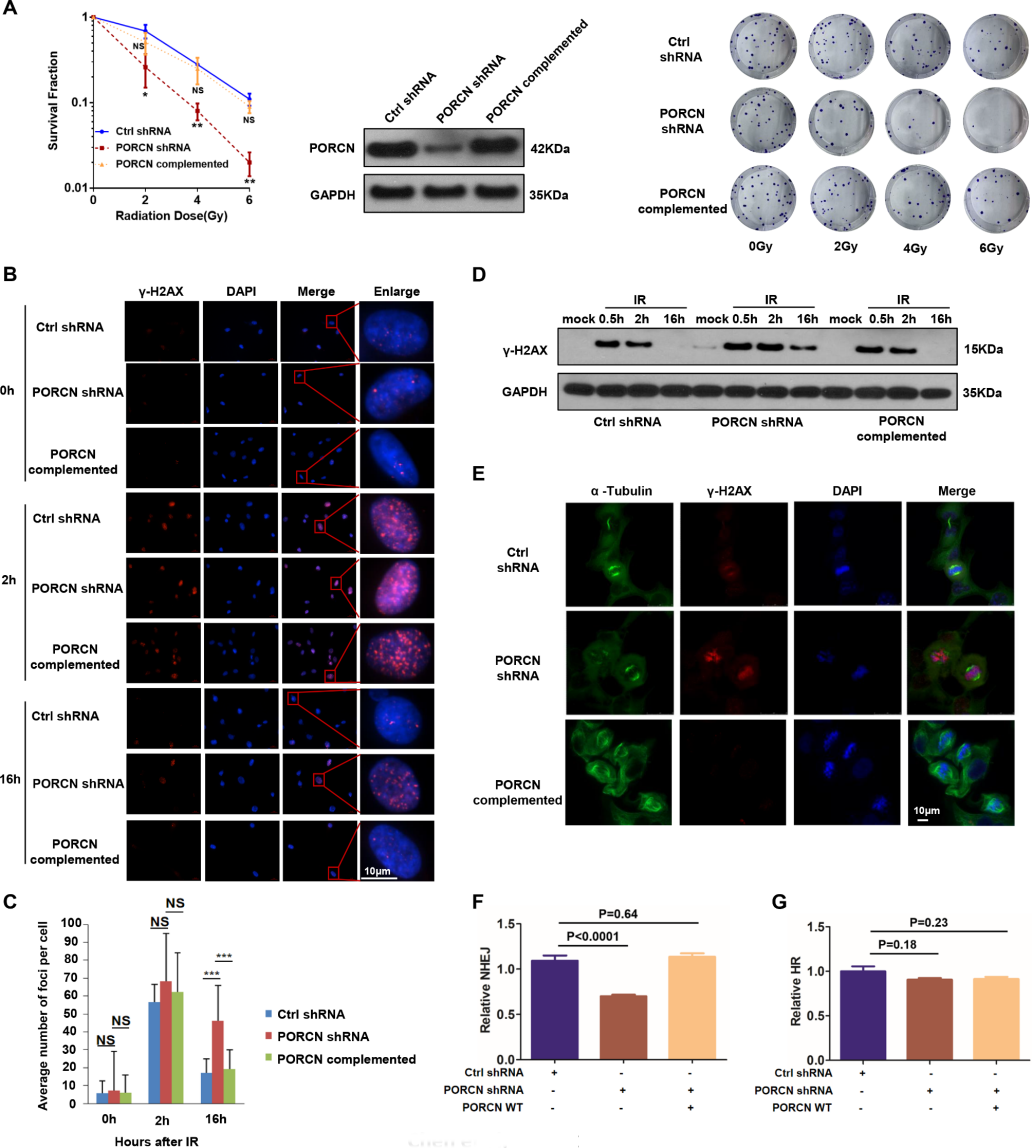
PORCN is essential for radiosensitivity and NHEJ. (**A**) Left: Surviving fractions were measured by the colony formation assay in HT1080 cells stably expressing control shRNA or PORCN shRNA with or without complementation with PORCN-WT. The data are presented as the means ± SDs of at least three independent experiments. *p* values were determined by two-way ANOVA. Middle: expression of PORCN measured by Western blot analysis. Right: representative colony formation assay images from each experimental group. (**B**) The indicated HT1080 cells were treated with or without IR. Sixteen hours after IR, the cells were fixed, stained with γ-H2AX and DAPI, and subjected to immunofluorescence microscopy. Scale bar: 10 μm. (**C**) The number of γ-H2AX foci was counted, and the average number of nuclear foci per cell is presented. The data are presented as the means ± SDs from three independent experiments. At least 50 cells were analysed per cell line/experiment in the IF experiments. *p* values were determined by two-way ANOVA. (**D**) The indicated HT1080 cells were mock-treated or irradiated with 5 Gy and collected at the indicated time points. Total cell lysates were harvested and subjected to Western blot analysis using the indicated antibodies. (**E**) The indicated HT1080 cells were treated with IR. Sixteen hours after IR, the cells were fixed, stained with anti-α-tubulin or anti-γ-H2AX antibodies and DAPI, and subjected to immunofluorescence microscopy. Representative confocal microscopy images of irradiated cells are shown. Scale bar: 10 μm. (**F**) EJ5-U2OS cells were transfected with the indicated plasmids or shRNAs and subjected to the NHEJ assay. The ratio of GFP-positive cells in the control group was set as “1.0”. The data are presented as the means ± SDs from three independent experiments and *p* values are presented. *p* values were determined by two-way ANOVA. (**G**) DR-U2OS cells were transfected with the indicated plasmids or shRNAs and subjected to the HR assay. The ratio of GFP-positive cells in the control group was set as “1.0”. The data are presented as the means ± SDs from three independent experiments and *p* values are presented. *p* values were determined by two-way ANOVA

To study whether PORCN is directly involved in the DDR, we treated cells with the PORCN inhibitor and measured the impact on radiosensitivity. We found that treatment with PORCN inhibitor LGK974 or ETC-159 caused hypersensitivity to IR in HCT116, HeLa, MCF-7 and MDA-MB-231 cells (Supplemental Fig. 1A-H). In addition, knocking down PORCN significantly increased radiosensitivity in HeLa and HT29 cells (Supplemental Fig. 2A-C). We also generated an shRNA-resistant PORCN construct and induced its expression in PORCN-knockdown HT1080 cells to assess complementation effects. We found that re-expression of wild-type PORCN (PORCN-WT) rescued the phenotype in PORCN-knockdown cells (Fig. 1A**)**, indicating that PORCN participates in regulating cellular radiosensitivity. We also evaluated IR-induced γ-H2AX focus formation, a classic marker for DNA DSB repair. As shown in Fig. 1B-D, knockdown of PORCN by shRNA resulted in prolonged existence of γ-H2AX, and reintroduction of PORCN-WT reversed this change. Confocal microscopy analysis of PORCN-knockdown cells showed the accumulation of γ-H2AX foci during mitosis (α-tubulin staining) (Fig. 1E), indicating that in the absence of PORCN, cells might not have completed DNA repair upon entering mitosis. This phenomenon was rescued when we reintroduced PORCN into cells (Fig. 1E).

To further investigate whether PORCN is involved in DNA damage repair, we assessed HR and NHEJ activity in DR-U2OS and EJ5-U2OS cells, respectively. We deleted endogenous PORCN in these cells using shRNA. As shown in Fig. 1F, PORCN-knockdown EJ5-U2OS cells displayed defective NHEJ compared to control shRNA cells. We also performed rescue experiments by reintroduction of PORCN-WT. As shown in Fig. 1F, reintroduction of PORCN-WT rescued the impairment of NHEJ. However, HR activity was not affected when PORCN was knocked down in DR-U2OS cells (Fig. 1G). These findings indicate that PORCN is required for the activation of NHEJ.

In addition, we used isogenic HT1080 cells (PORCN-WT or PORCN-null) [34] to test whether PORCN is required for DNA damage repair and genomic stability. The comet assay demonstrated that DNA repair was significantly delayed in PORCN-null cells (Supplemental Fig. 3A and 3B). Giemsa staining showed significantly more multinucleation and micronucleation in PORCN-null cells than in PORCN-WT cells (Supplemental Fig. 3C and 3D). We also checked whether PORCN is involved in the initiation of the G2/M checkpoint in response to DNA damage. Supplemental Fig. 3E and 3 F show that there was no significant difference in G2/M checkpoint activation between PORCN-WT and PORCN-null cells. Furthermore, we found that PORCN-null cells showed normal ATM activation (serine 1981 autophosphorylation in ATM) in response to IR (Supplemental Fig. 4A) compared to control cells. We also found that there was no difference in IR-induced pATM(S1981) focus formation between PORCN-WT and PORCN-null cells (Supplemental Fig. 4B).

Because PORCN plays a critical role in the Wnt/β-catenin signalling pathway, we then investigated whether the PORCN-mediated Wnt pathway is affected by IR. To this end, we tested Wnt/β-catenin signalling activation in isogenic HT1080 cells in response to IR. As an essential Wnt coreceptor for the Wnt/β-catenin signalling pathway, LRP6 must be phosphorylated for Wnt/β-catenin signalling activation upon Wnt ligand binding [35–37]. As expected, PORCN-null cells displayed a reduced level of LRP6 phosphorylation and consequently a lower level of cytosolic free β-catenin than their wild-type counterparts (Supplemental Fig. 4C). However, neither PORCN-WT nor PORCN-null cells showed alterations in the Wnt pathway in response to IR. These data indicate that PORCN-dependent Wnt signalling is not affected by IR-induced DNA damage.

### The NLS domain is essential for PORCN to enter the nucleus

**Fig. 2:**
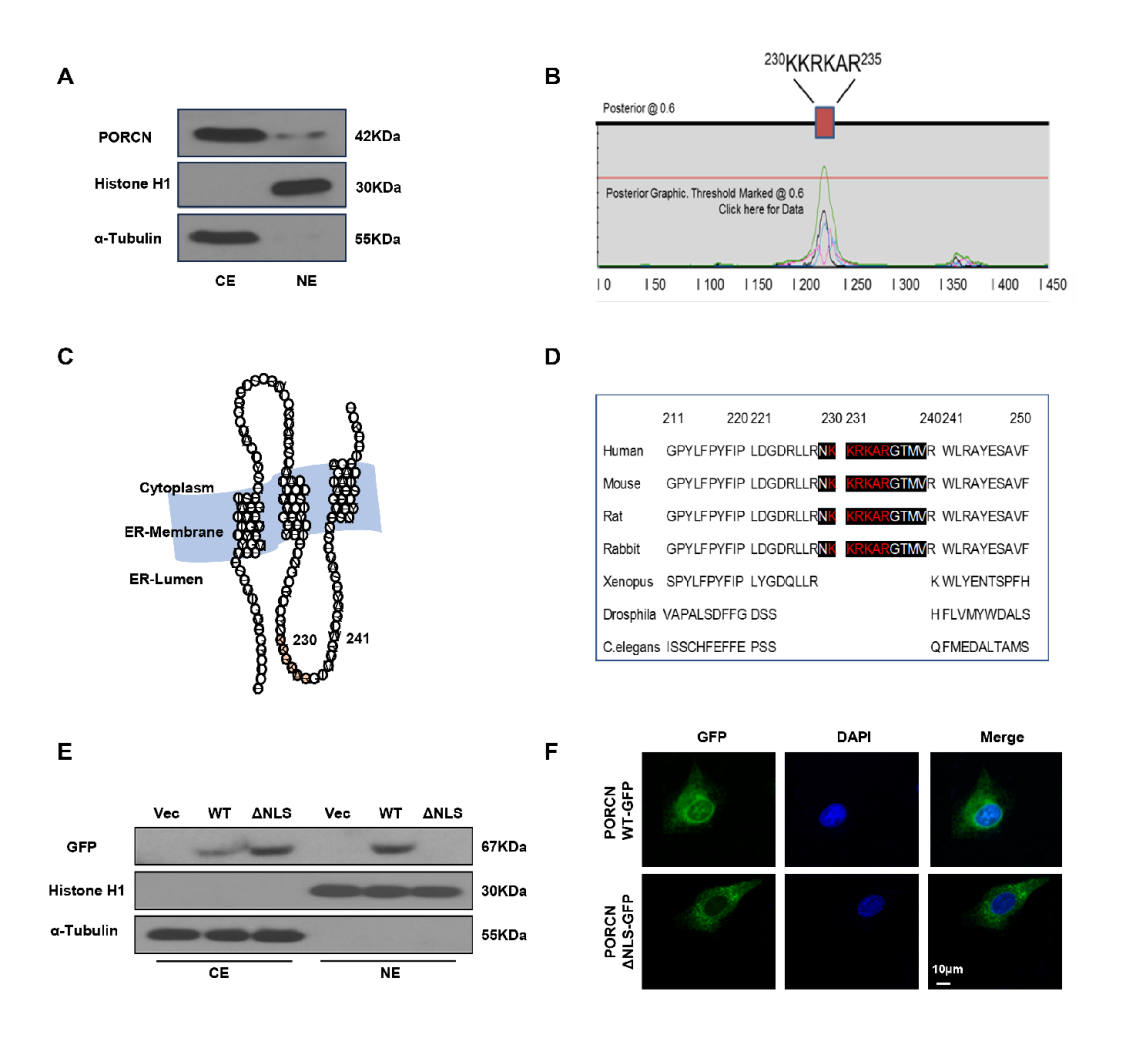
The NLS domain is essential for PORCN to enter the nucleus. (**A**) Exponentially growing HT1080 cells were harvested, and cytoplasmic and nuclear fractions were obtained for Western blot analysis using the anti-PORCN antibody. α-tubulin and Histone H1 were used as subcellular compartment markers for the cytoplasm and nucleus, respectively. (**B**) The primary sequence of PORCN was run through a nuclear localization sequence (NLS) predictor (www.moselab.csb.utoronto.ca/NLStradamus), and the KKRKAR sequence spanning residues 230–235 was predicted as a potential NLS. (**C**) Schematic of the NLS region in the ER lumen. (**D**) Sequence conservation analysis was performed across species. (**E**) Cytoplasmic and nuclear extracts from HT1080 cells expressing vector control, GFP-tagged PORCN-WT or PORCN-ΔNLS were harvested and subjected to SDS-PAGE and immunoblotting with anti-GFP, anti-histone H1, and anti-α-tubulin antibodies. (**F**) Confocal microscopic images of localization patterns of GFP-tagged PORCN-WT (left) or PORCN-ΔNLS (right) in HT1080 cells transiently transfected with the corresponding plasmids. Scale bar: 10 μm

Because most biochemical events related to the DDR occur within the nucleus, we tested the possibility that there might be a nuclear fraction of PORCN. We separated the nuclear and cytosolic fractions of HT1080 cell lysates and performed immunoblotting with an anti-PORCN antibody. We found that there was a small portion of PORCN in the nuclear fraction (Fig. 2A). We then ran the primary sequence of PORCN through a nuclear localization sequence (NLS) predictor and identified the KKRKAR sequence spanning amino acid residues 230–235 as a potential NLS (Fig. 2B). This region is located on the ER lumen, as shown by the schematic in Fig. 2C. Sequence conservation analysis showed that this putative NLS was found only in mammalian species (Fig. 2D). To determine whether the NLS domain is required for nuclear localization, we generated a GFP-tagged, NLS-deleted (ΔNLS) construct of PORCN and induced its expression in HT1080 cells. PORCN-WT and PORCN-ΔNLS were both expressed in the cytosolic portion of the cell lysates. However, PORCN-ΔNLS failed to localize to the nucleus (Fig. 2E). Confocal microscopy showed that GFP-tagged PORCN-WT displayed clear nuclear membrane localization (a ring shape) in HT1080 and HT29 cells. However, ΔNLS did not display any nuclear signal (Fig. 2F and Supplemental Fig. 5A).

To investigate whether the NLS domain affected PORCN-mediated Wnt activity, we measured the levels of phosphorylated LRP6 and free β-catenin in HT1080 cells with PORCN knockdown with or without complementation with PORCN-WT or PORCN-ΔNLS. As shown in Supplemental Fig. 5B, knocking down PORCN resulted in the depletion of phosphorylated LRP6 and free β-catenin. However, we found that WT or ΔNLS complementation in PORCN knockdown cells could rescue this change, indicating that the NLS domain is not required for PORCN-mediated Wnt signalling.

### The NLS domain is important for PORCN’s role in radiosensitivity and NHEJ

**Fig. 3:**
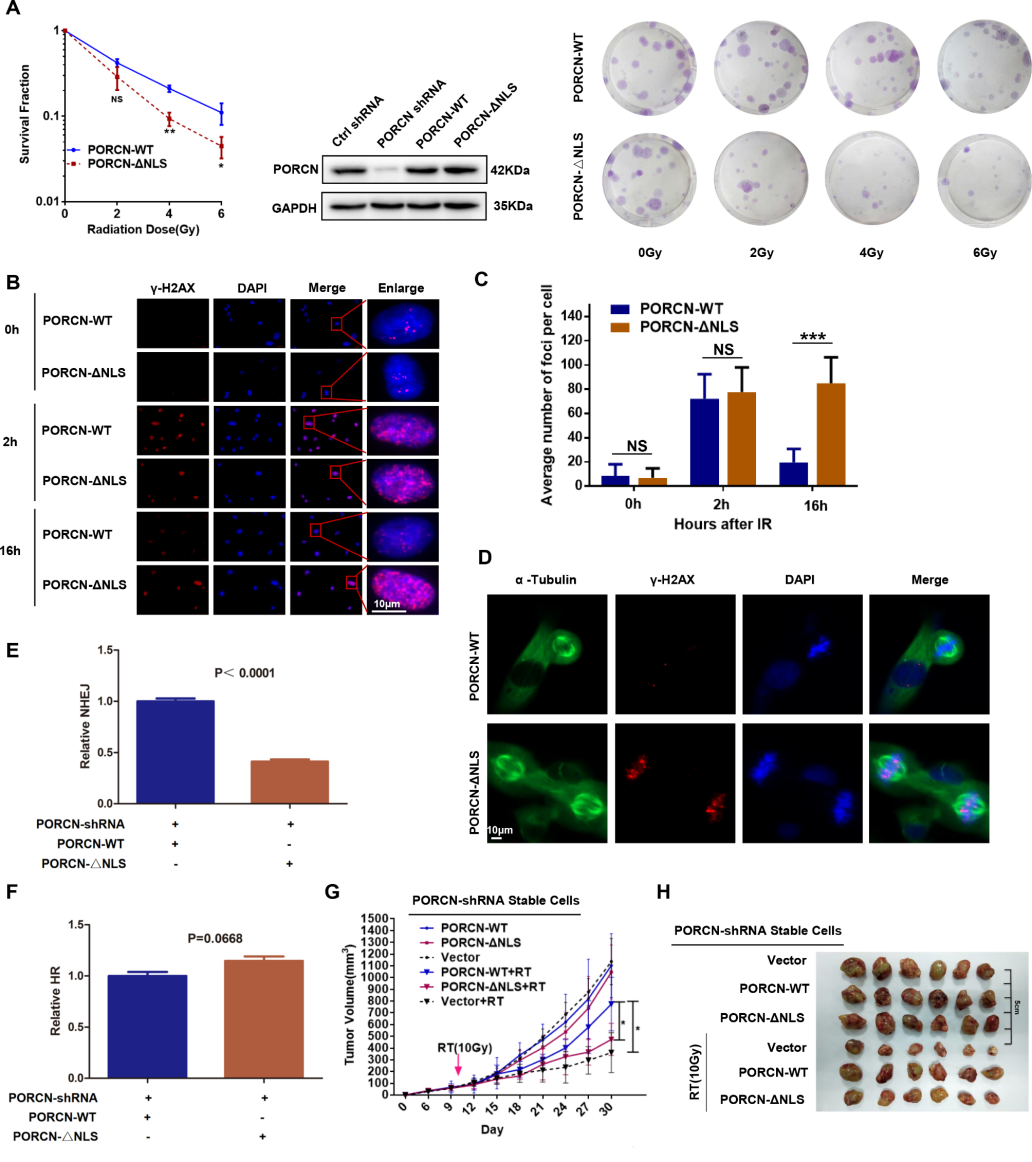
The NLS domain of PORCN is required for DDR activation. (**A**) PORCN-knockdown HT1080 cells with PORCN-WT or PORCN-ΔNLS complementation were obtained. The percentage of surviving cells after IR measured by the colony formation assay is shown on the left. The data are presented as the means ± SDs from three independent experiments, and *p* values determined by Student’s t test are shown. Middle: knockdown of PORCN-WT and PORCN-NLS and rescue of PORCN-WT and PORCN-NLS protein expression levels were validated with an anti-PORCN antibody. Representative images of the colony formation assay are shown on the right. (**B-C**) HT1080 cells with PORCN-WT or PORCN-ΔNLS complementation were treated with or without IR. Sixteen hours after IR, the cells were fixed and subjected to immunofluorescence staining with DAPI and anti-γ-H2AX antibodies. Confocal microscopy images of irradiated cells are shown in B. Scale bar: 10 μm. The number of γ-H2AX foci was counted, and the average number of nuclear foci per cell is presented in C. The data are presented as the means ± SDs from three independent experiments. At least 50 cells were analysed per cell line/experiment in the IF experiments. *p* values determined by Student’s t test are shown. (**D**) HT1080 cells with PORCN-WT or PORCN-ΔNLS complementation were treated with IR. Sixteen hours after IR, the cells were fixed and stained with DAPI or subjected to immunofluorescence staining with anti-α-tubulin or anti-γ-H2AX antibodies. Confocal microscopy images of irradiated cells are shown. Scale bar: 10 μm. (**E-F**) EJ5-U2OS and DR-U2OS cells were transfected with the indicated plasmids or shRNAs and subjected to NHEJ (**E**) and HR (**F**) assays, respectively. The ratio of GFP-positive cells in the PORCN-WT group was set as “1.0”. The data are presented as the means ± SDs from three independent experiments and *p* values determined by Student’s t test are shown. (**G**) Tumour growth curves after IR in xenograft mice are shown. Tumour volumes were measured every 3 days. All data represent the means ± SDs. (**H**) Xenografts obtained from BALB/c nu/nu mice in the experimental groups

To investigate the function of nuclear PORCN (nPORCN), we first established stable cell lines with PORCN-WT and PORCN-ΔNLS-complementation from the PORCN-knockdown HT1080 cell line. The colony formation assay showed that PORCN-ΔNLS cells were more sensitive to IR than PORCN-WT cells (Fig. 3A). We also found that the expression of γ-H2AX and the number of γ-H2AX foci remained high in PORCN-ΔNLS cells 16 h after IR, whereas in PORCN-WT cells, they had already returned to a normal level in HT1080 cells (Fig. 3B **and C).** In HT29 cells, the expression of γ-H2AX were also remained high in PORCN-ΔNLS cells 16 h after IR (Supplemental Fig. 5C). In addition, we found that PORCN-ΔNLS cells showed accumulation of γ-H2AX foci in mitosis (Fig. 3D). Furthermore, we used EJ5-U2OS and DR-U2OS cells with PORCN knockdown and reintroduction of PORCN-WT or PORCN-ΔNLS to check NHEJ and HR activity. We found that PORCN-ΔNLS cells had a 45% decrease in NHEJ efficiency compared to PORCN-WT cells (Fig. 3E). However, PORCN-ΔNLS did not have any impact on HR efficiency compared to WT cells (Fig. 3F). We also found significantly more multinucleation in PORCN-ΔNLS cells than in PORCN-WT cells (Supplemental Fig. 5D). Furthermore, PORCN-ΔNLS cells showed impaired DNA-PKcs activation (serine 2056 phosphorylation in DNA-PKcs) in response to IR (Supplemental Fig. 5E).

To determine the role of nPORCN in radiosensitivity in vivo, we injected PORCN-knockdown cells with a vector, PORCN-WT, or PORCN-ΔNLS complementation into xenograft nude mice. Similarly to vector xenografts, PORCN-ΔNLS xenografts showed significantly more IR-induced tumour growth delay than PORCN-WT xenografts **(**Fig. 3G and H**)**. Taken together, our results demonstrate that the NLS domain is critical for nPORCN’s function in the DDR.

### PORCN NLS knockout cells show similar DDR defects to PORCN knockout cells

**Fig. 4:**
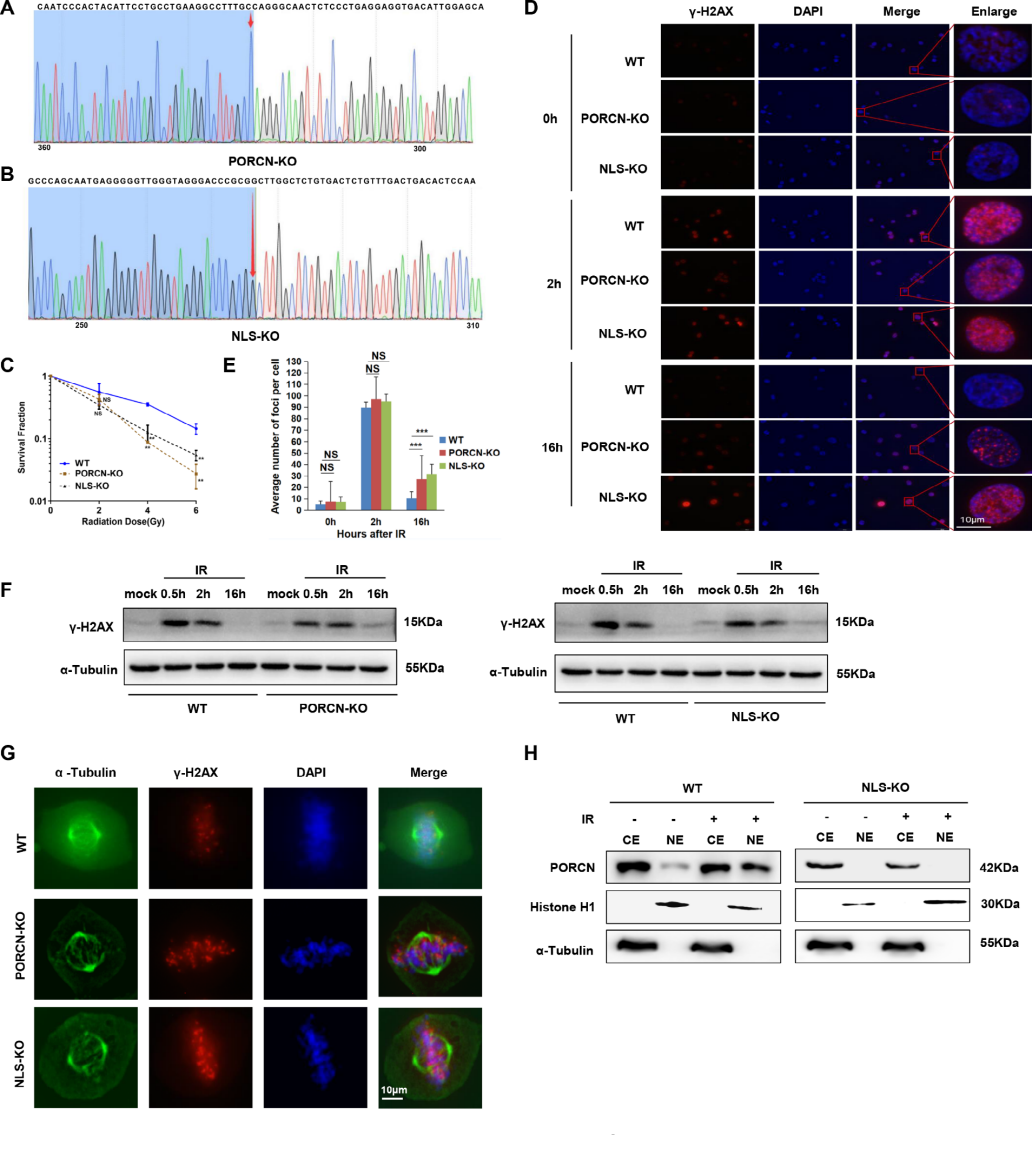
PORCN-KO and NLS-KO cells show impaired DNA damage repair capacity. (**A**) Sanger sequencing of HT1080 PORCN-KO cells constructed using CRISPR/Cas9. (**B**) Sanger sequencing of NLS-KO cells constructed using CRISPR/Cas9. (**C**) The percentage of surviving HT1080 PORCN-WT, PORCN-KO, and NLS-KO cells was measured by the colony formation assay. The data are presented as the means ± SDs from three independent experiments, and *p* values were determined by two-way ANOVA. (D-E) The indicated isogenic cells were treated with or without 5 Gy IR. Sixteen hours after IR, the cells were fixed, stained with γ-H2AX and DAPI, and subjected to immunofluorescence microscopy. Representative confocal microscopy images are shown in (**D**). Scale bar: 10 μm. The number of γ-H2AX foci was counted, and the average number of nuclear foci per cell is presented in (**E**). The data are presented as the means ± SDs from three independent experiments. (**F**) The indicated HT1080 cells were mock-treated or irradiated with 5 Gy and collected at the indicated time points. Total cell lysates were harvested and subjected to Western blot analysis using the indicated antibodies. (**G**) The indicated HT1080 cells were treated with IR (5 Gy). Sixteen hours after IR, the cells were fixed, stained with anti-α-tubulin or γ-H2AX antibodies and DAPI, and subjected to immunofluorescence microscopy. Representative confocal microscopy images of irradiated cells are shown. Scale bar: 10 μm. (**H**) Cytoplasmic and nuclear extracts from HT1080 PORCN-WT or NLS-KO cells were exposed to IR (5 Gy). After 2 h, the cells were subjected to SDS-PAGE and immunoblotted with anti-PORCN, anti-Histone H1, and anti-α-tubulin antibodies

To further examine the role of PORCN NLS in the DDR, we generated PORCN knockout (KO) and NLS knockout (NLS-KO) HT1080 cell lines using CRISPR-Cas9 (Fig. 4A **and B**). Consistent with the impact of PORCN-KO cells, NLS-KO cells exhibited heightened sensitivity to IR (Fig. 4C). Furthermore, NLS-KO cells displayed prolonged presence of γ-H2AX and accumulation of γ-H2AX foci during mitosis (Fig. 4D-G). Taken together, our results demonstrate that the NLS domain is critical for nPORCN’s function in the DDR.

Given that Gao. et al. demonstrated that PORCN facilitates the palmitoylation of Wnt3a [38], we aimed to explore the impact of the NLS domain on Wnt3a palmitoylation in response to IR. As depicted in Supplemental Fig. 6A, the analysis revealed no discernible disparity in Wnt3a palmitoylation between WT and NLS KO cell lines, regardless of whether the cells were subjected to IR. This finding suggests that the role of nPORCN in the DDR is independent of its involvement in the Wnt signalling pathway.

The activation mechanism of nPORCN in response to IR is critical to understanding how nPORCN participates in the DDR; hence, we tested whether IR induces PORCN translocation into the nucleus. Figure 4H and Supplemental Fig. 6B shows that nPORCN levels were increased in response to IR.

### Identification of a protein complex of nPORCN in response to IR-induced DNA damage

**Fig. 5:**
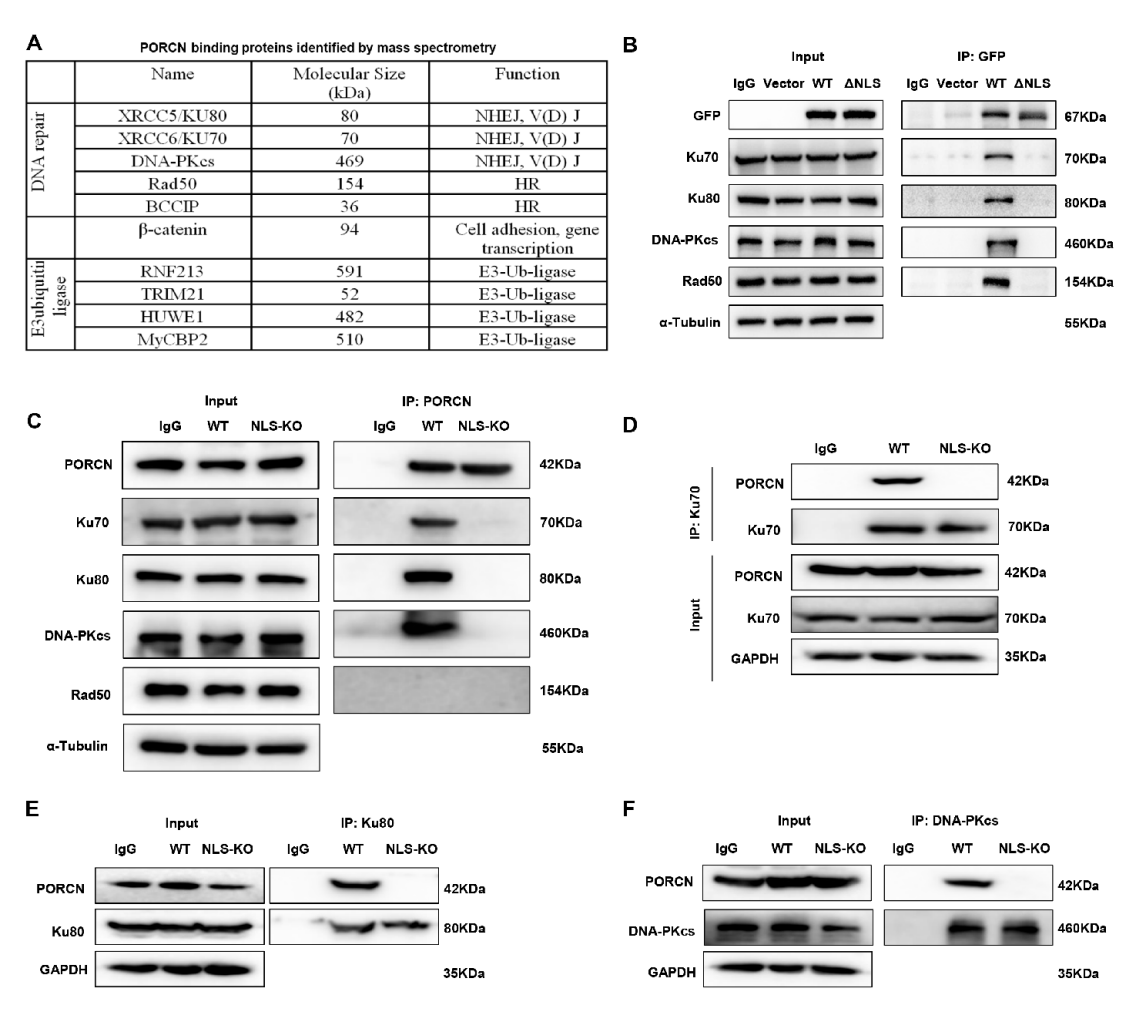
Identification of nPORCN-interacting proteins. (**A**) List of the top PORCN-binding proteins identified by mass spectrometry. (**B**) HT1080 cells were transiently transfected with GFP-tagged PORCN-WT or PORCN-ΔNLS. Forty-eight hours after transfection, whole cell lysates were harvested and immunoprecipitated with an anti-GFP antibody. The immunoprecipitation products were subjected to SDS-PAGE and probed with anti-GFP, anti-Ku70, anti-Ku80, anti-DNA-PKcs, anti-Rad50, or anti-α-tubulin antibodies. (**C**) HT1080 PORCN-WT or NLS-KO cells were immunoprecipitated with an anti-PORCN antibody. The immunoprecipitation products were subjected to SDS-PAGE and probed with anti-PORCN, anti-Ku70, anti-Ku80, anti-DNA-PKcs, anti-Rad50, or anti-α-tubulin antibodies. (**D**) PORCN-WT or NLS-KO HT1080 cells were immunoprecipitated with an anti-Ku70 antibody. The immunoprecipitation products were subjected to SDS-PAGE and probed with an anti-PORCN antibody. (**E**) PORCN-WT or NLS-KO cells were immunoprecipitated with an anti-Ku80 antibody. The immunoprecipitation products were subjected to SDS-PAGE and probed with an anti-PORCN antibody. (**F**) PORCN-WT or NLS-KO HT1080 cells were immunoprecipitated with an anti-DNA-PKcs antibody. The immunoprecipitation products were subjected to SDS-PAGE and probed with an anti-PORCN antibody

To investigate how nPORCN regulates the DDR, we aimed to identify PORCN-interacting proteins in the nucleus by mass spectrometry. To achieve this goal, we conducted a coimmunoprecipitation (co-IP) experiment in PORCN-WT or PORCN-ΔNLS HT1080 cells. Exogenous proteins were immunoprecipitated, and the IP products were separated by electrophoresis, stained with silver (Supplemental Fig. 7A), and analysed by LC-ESI-MS/MS. Proteins were compared for changes before and after IR between WT and ΔNLS. The top proteins identified included five DNA repair proteins, four E3-Ub ligases, and β-catenin (Fig. 5A).

To validate the interaction between nPORCN and DNA repair proteins, we conducted co-IP experiments in HT1080 cells expressing GFP-tagged PORCN-WT or PORCN-ΔNLS. We found that in the immunoprecipitates from PORCN-WT cells, DNA-PKcs, Ku70 and Ku80 were detectable (Fig. 5B). However, the immunoprecipitates from PORCN-ΔNLS cells did not contain any of DNA-PK complexes (Fig. 5B). Furthermore, Rad50 was not observed in the immunoprecipitated samples (Fig. 5B). Additionally, through the utilization of NLS-KO isogenic cell lines, we were able to validate the interaction between the endogenous PORCN ΔNLS and the associated proteins in comparison to the WT through co-immunoprecipitation **(**Fig. 5C-F). DNA-PKcs, Ku70, and Ku80 were detectable in the immunoprecipitates from PORCN-WT cells but not in the immunoprecipitates from NLS-KO cells. These findings provide evidence that nPORCN interacts with the DNA-PKcs/Ku70/Ku80 complex, and this interaction relies on the NLS domain.

To investigate whether PORCN and Ku70 interact directly with each other, we also performed an in vitro co-IP assay using purified PORCN and GST-Ku70 proteins. As shown in Supplemental Fig. 7B, the purified PORCN protein could interact with GST-Ku70.

### Ku70 is palmitoylated in response to IR in an nPORCN-dependent manner

**Fig. 6:**
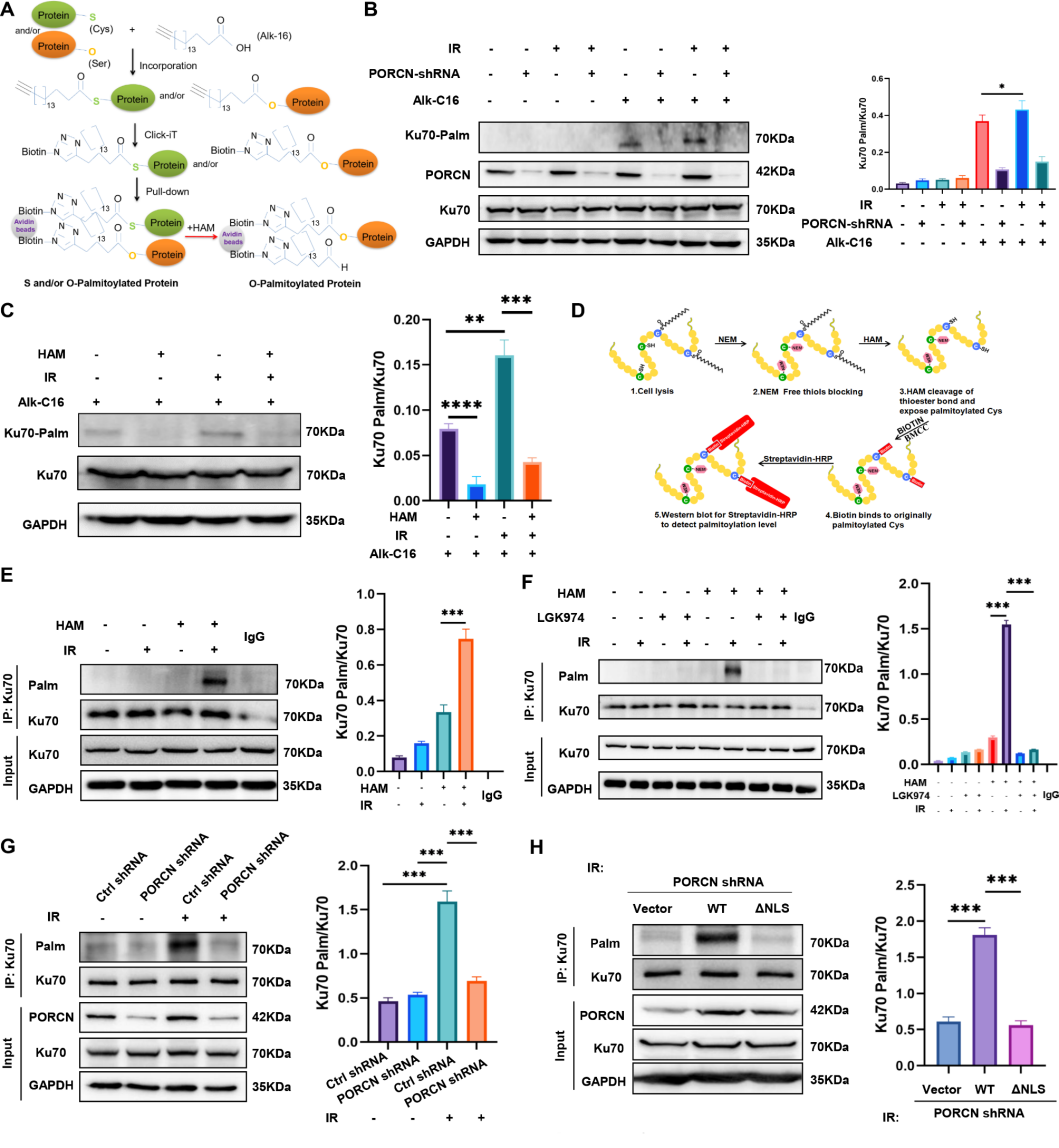
Ku70 is *S*-palmitoylated in response to IR in an nPORCN-dependent manner. (**A**) Illustration of the modified Click-iT assay used to detect *S*- and *O*-palmitoylation. (**B**) Control shRNA or PORCN shRNA HT1080 cells were cultured in medium containing palmitic acid-azide for 6 h. The cells were subjected to mock or IR treatment and prepared for the Click-iT assay. Palmitoylated proteins were precipitated using streptavidin Sepharose beads and probed with the indicated antibodies. Quantitative data from HT1080 cells are shown. The data are presented from three independent experiments. (**C**) HT1080 cells cultured in medium containing palmitic acid-azide for 6 h were subjected to mock or IR treatment. The cells were then prepared for the Click-iT assay in the presence or absence of HAM. Palmitoylated proteins were precipitated using streptavidin Sepharose beads and probed with the indicated antibodies. Quantitative data from HT1080 cells are shown. The data are presented from three independent experiments. (**D**) Illustration of the ABE method used to identify *S-*palmitoylated proteins. (**E**) HT1080 cells were subjected to mock or IR treatment. Ku70 was immunoprecipitated and subjected to the ABE assay in the presence or absence of HAM. The palmitoylated proteins were probed with the Streptavidin-HRP antibody. Quantitative data from HT1080 cells are shown. The data are presented from three independent experiments. (**F**) HT1080 cells cultured in medium containing LGK974 were subjected to mock or IR treatment. Ku70 was immunoprecipitated and subjected to the ABE assay in the presence or absence of HAM. Palmitoylated proteins were blotted with a Streptavidin-HRP antibody. Quantitative data from HT1080 cells are shown. The data are presented from three independent experiments. (**G**) Stable HT1080 cells were subjected to mock or IR (5 Gy) treatment. Ku70 was immunoprecipitated and subjected to the ABE assay in the presence of HAM. Palmitoylated proteins were immunoblotted with a Streptavidin-HRP antibody. Quantitative data from HT1080 cells are shown. The data are presented from three independent experiments. (**H**) Stable HT1080 PORCN shRNA cells transfected with empty vector, PORCN-WT or PORCN-ΔNLS were subjected to mock or IR (5 Gy) treatment. Ku70 was immunoprecipitated and processed for the ABE assay in the presence of HAM. Palmitoylated proteins were blotted with a Streptavidin-HRP antibody. Quantitative data from HT1080 cells are shown. The data are presented from three independent experiments

Since PORCN is known as an *O-*acyltransferase, it is possible that Ku70 might be its palmitoylation target. To test this possibility, we used the Click-iT labelling assay, which can be used to identify both *S-* and *O-*palmitoylated proteins. As illustrated in Fig. 6A, we used a modified Click-iT labelling method to distinguish the two types of palmitoylated proteins. We labelled immunoprecipitated Ku70 with Click-iT palmitic acid-azide (Alk-16) and allowed them both to react with biotin-alkyne. The biotin alkyne-azide–palmitic protein complex was pulled down by streptavidin. Palmitoylated proteins (both *S-* and *O-*palmitoylated proteins) can be detected by Western blot. When a protein is exposed to hydroxylamine (HAM), the *S-*palmitoyl group is removed, and only *O-*palmitoylated proteins can be detected. As shown in Fig. 6B, palmitoylated Ku70 was detectable in WT cells but not in PORCN-shRNA cells. Moreover, we observed an increase in Ku70 palmitoylation in cells exposed to IR. Surprisingly, following HAM treatment, we found that the palmitoylation of Ku70 was diminished, indicating that Ku70 is *S-*palmitoylated (Fig. 6C). Since PORCN was previously reported to be an *O-*acyltransferase [18], we suspected that nPORCN might have different enzymatic activity (*S-*acyltransferase) than its cytoplasmic component (*O-*acyltransferase). To validate this hypothesis, we used the acyl-biotinyl exchange (ABE) method **(**Fig. 6D**)**, which only identifies *S-*palmitoylated proteins, to study the palmitoylation of Ku70. As shown in Fig. 6E, Ku70 *S-*palmitoylation was not detectable in unperturbed cells. However, there was a strong signal in cells treated with IR, indicating IR-induced Ku70 palmitoylation. Furthermore, IR-induced Ku70 palmitoylation was not observed in cells in the presence of LGK974 (Fig. 6F**)** or PORCN-shRNA **(**Fig. 6G). These data demonstrate that Ku70 *S-*palmitoylation is dependent on PORCN activity. To study whether nPORCN is required for Ku70 palmitoylation, HT1080 cells stably expressing PORCN-WT, PORCN-∆NLS or an empty vector were used for the ABE assay. We found that PORCN-∆NLS completely ablated IR-induced Ku70 palmitoylation (Fig. 6H) compared to PORCN-WT cells. These data indicate that the NLS domain of PORCN is essential for Ku70 *S-*palmitoylation. Since the mass spectrometry results showed that nPORCN interacted with the DNA-PKcs/Ku70/Ku80 complex, we also investigated whether DNA-PKcs or Ku80 could also be palmitoylated by PORCN. As shown in Supplemental Fig. 7C and D, neither DNA-PKcs nor Ku80 was palmitoylated by PORCN.

### IR induces palmitoylation of five cysteine residues of Ku70

**Fig. 7:**
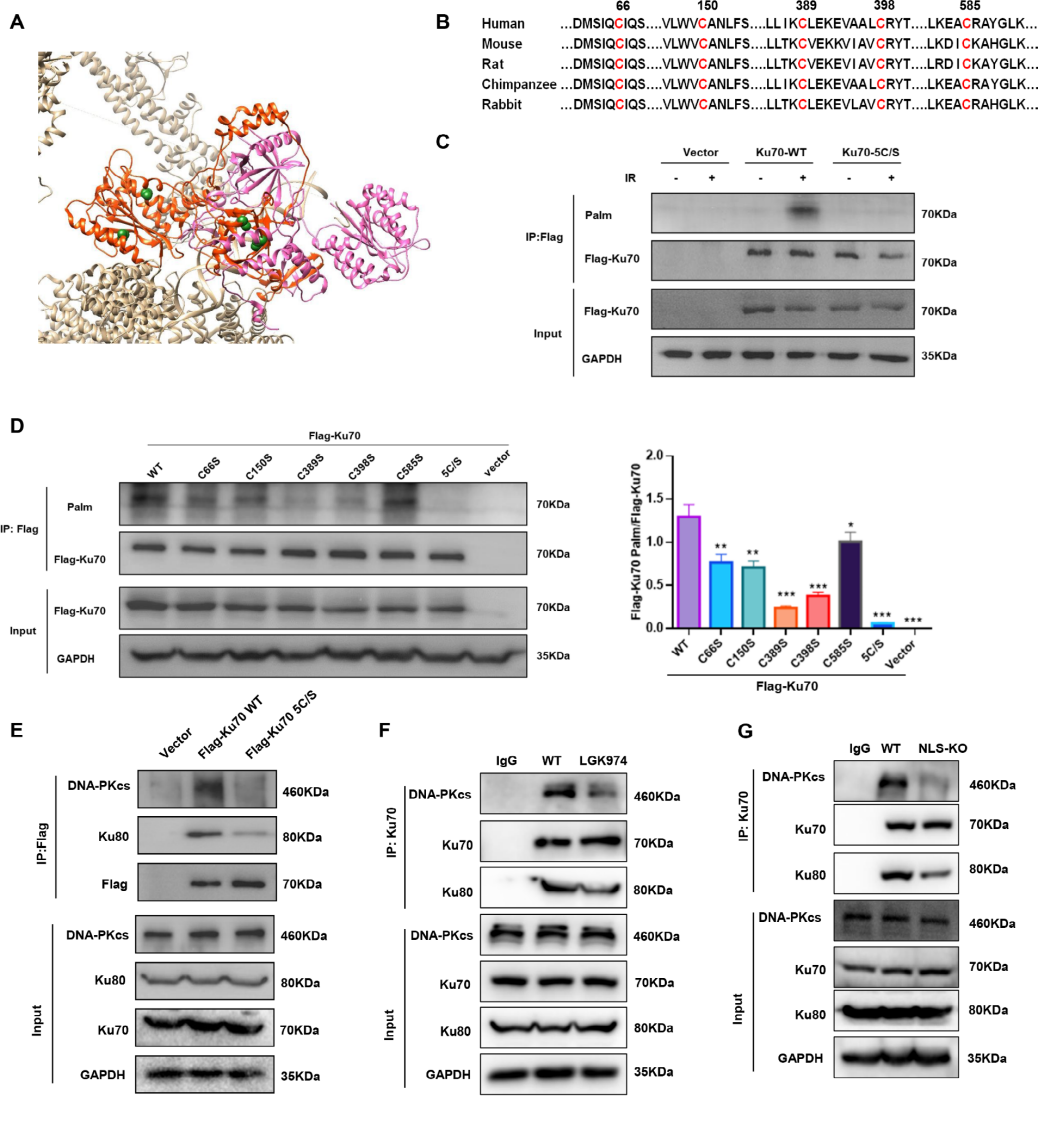
Identification of Ku70 palmitoylation sites. (**A**) A molecular dynamics simulation-derived structure of Ku70 (in red) with C66, C150, C389, C398 and C585 (in cyan) bound to Ku80 (in pink). (**B**) Sequence conservation analysis of Ku70 cysteine residues. (**C**) HT1080 cells transfected with empty vector, Flag-tagged Ku70-WT or Ku70-5 C/S were subjected to mock or IR treatment. Flag-Ku70 was immunoprecipitated and processed for the ABE assay in the presence of HAM. Palmitoylated proteins were immunoblotted with a Streptavidin-HRP antibody. (**D**) HT1080 cells transfected with empty vector, Flag-tagged Ku70-WT, Ku70-C66S, PORCN-C150S, Ku70-C389S, Ku70-C398S, Ku70-C585S or Ku70-5 C/S were subjected to IR treatment. Flag-Ku70 was immunoprecipitated and processed for the ABE assay in the presence of HAM. Palmitoylated proteins were immunoblotted with a Streptavidin-HRP antibody. Quantitative data from HT1080 cells are shown. The data are presented from three independent experiments and *p* values were determined by two-way ANOVA. (**E**) HT1080 cells were transfected with flag-tagged Ku70-WT or Ku70-5 C/S plasmids and irradiated (5 Gy). Two hours after IR, whole-cell extracts were collected and subjected to immunoprecipitation with an anti-Flag antibody. The immunoprecipitates were subjected to Western blot. (**F**) HT1080 cells were treated with DMSO or LGK974 (1 nM for 24 h) and then exposed to IR (5 Gy). Two hours after IR, whole-cell extracts were collected and subjected to immunoprecipitation with an anti-Ku70 antibody. The immunoprecipitates were subjected to Western blot. (**G**) PORCN WT or NLS-KO HT1080 cells were exposed to IR (5 Gy). Two hours after IR, whole-cell extracts were collected and subjected to immunoprecipitation with an anti-Ku70 antibody. The immunoprecipitates were subjected to Western blot

To identify Ku70 *S*-palmitoylation sites, we analysed the Ku70 sequence and found cysteine (Cys) residues located at positions 66, 150, 389, 398 and 585 (Fig. 7A**)**. These cysteines are well conserved in mammalian species (Fig. 7B**)**. We individually mutated each of the Cys residues, as well as all five, to serine (C66S, C150S, C389S, C398S, C585S, and 5 C/S). As shown in Fig. 7C, IR-induced Ku70 *S-*palmitoylation was ablated when the cysteine residues were mutated to serine (5 C/S). We then examined which mutant influences the palmitoylation signal. Mutation of four cysteine residues (66, 150, 389, and 398) to serine partially reduced the palmitoylation signal of Ku70. Moreover, we noticed that there was a minor reduction in the palmitoylation of the C585S mutant **(**Fig. 7D-E). The C585 mutation reduced palmitoylation by 22.5%, and the C66, C150, C389 and C398 mutations reduced palmitoylation by 41.1%, 45.0%, 81.4% and 70.6%, respectively.

Since Ku70 interacts with DNA-PKcs and Ku80, we then determined the effect of Ku70 *S*-palmitoylation on DNA-PKcs/Ku70/Ku80 complex formation. We conducted co-IP experiments in HT1080 cells expressing Flag-tagged Ku70-WT or Ku70-5 C/S. We found that the interaction of the complex proteins was reduced in Ku70-5 C/S cells (Fig. 7F). We also demonstrated that the Ku70 5 C/S mutant significantly affects DNA-PK autophosphorylation (Supplemental Fig. 8A). As shown in Fig. 7G **and H**, less DNA-PKcs immunoprecipitated with Ku when PORCN was inhibited by LGK974 or the PORCN NLS was depleted. These results demonstrate that the formation of the DNA-PKcs/Ku70/Ku80 complex is dependent on PORCN-mediated Ku70 palmitoylation.

We also investigated whether DNA-PKcs/Ku70/Ku80 complex could be also essential for the nuclear retention of nuclear PORCN. We observed that despite disrupting DNA-PK complex formation by either Ku70 knockdown or using the Ku70 5/S mutant cells, which affects Ku70-mediated S-palmitoylation critical for complex formation, the nuclear expression of PORCN remained unchanged (Supplemental Fig. 8B-C). These results suggest that the formation of the DNA-PK complex does not directly influence the nuclear retention of PORCN.

### Ku70 palmitoylation is important for NHEJ and radiosensitivity

**Fig. 8:**
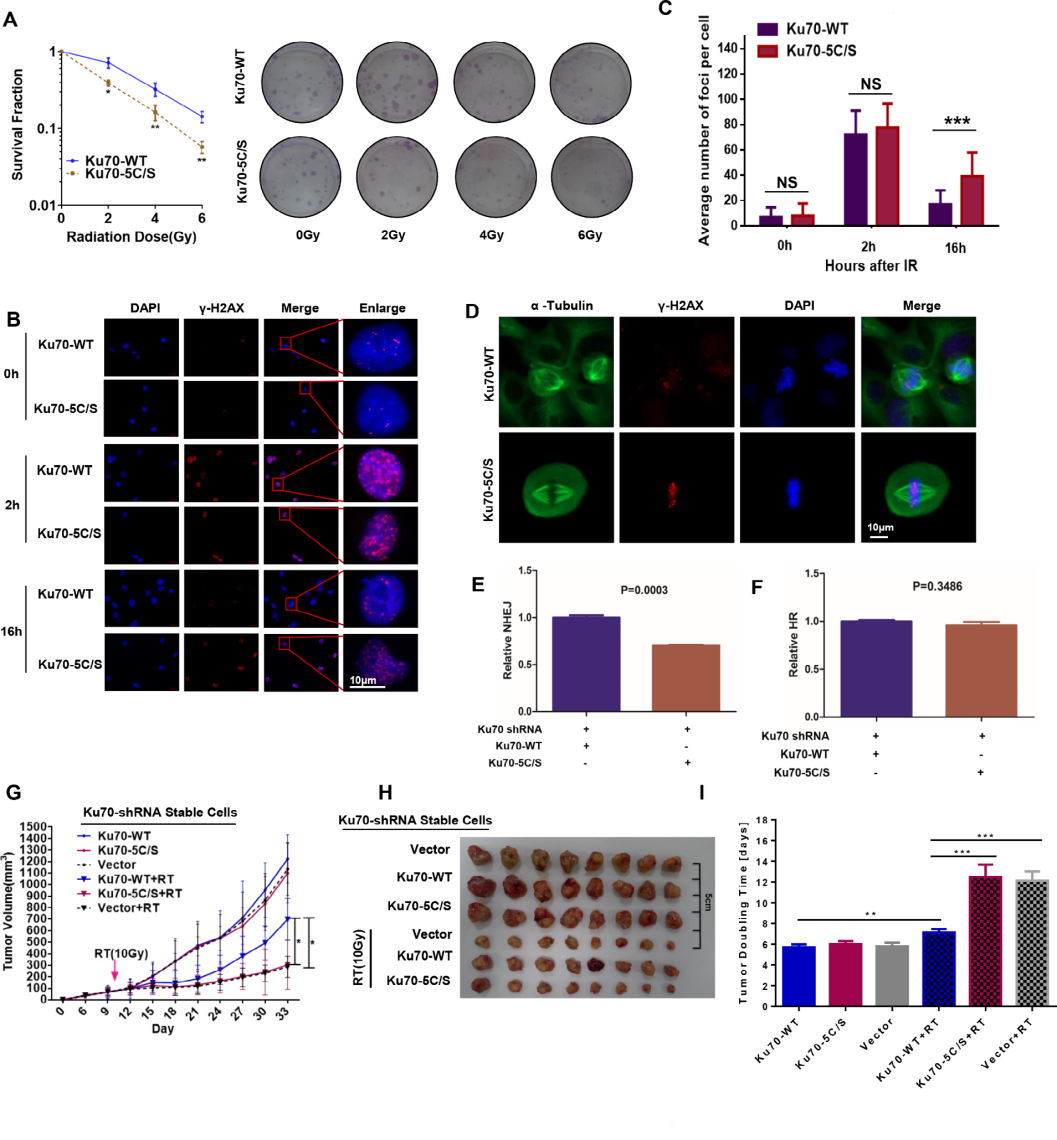
nPORCN-dependent Ku70 *S-* palmitoylation is critical for NHEJ and radiosensitivity. (**A**) HT1080 cells were stably knocked down with Ku70 shRNA and complemented with Ku70-WT or Ku70-5 C/S. The percentage of surviving cells after IR measured by the colony formation assay is shown on the left. Representative images of the colony formation assay are shown on the right. The data are presented as the means ± SDs from three independent experiments and *p* values determined by Student’s t test are shown. (B-C) HT1080 cells with Ku70-WT or Ku70-5 C/S- complementation were subjected to mock or IR (5 Gy) treatment. Sixteen hours after IR, the cells were fixed, subjected to immunofluorescence staining with an anti-γ-H2AX antibody, stained with DAPI and visualized under a microscope. Confocal microscopy images of irradiated cells are shown in (**B**). Scale bar: 10 μm. The number of γ-H2AX foci was counted, and the average number of nuclear foci per cell is presented. The data are presented as the means ± SDs from three independent experiments. *p* values determined by Student’s t test are shown. At least 50 cells were analysed per cell line/experiment in the IF experiments. (**D**) HT1080 cells with Ku70-WT or Ku70-5 C/S complementation were treated with IR (5 Gy). Sixteen hours after IR, the cells were fixed and subjected to immunofluorescence staining with anti-α-tubulin or γ-H2AX antibodies and DAPI staining. Confocal microscopy images of irradiated cells are shown. Scale bar: 10 μm. (E and F) EJ5-U2OS and DR-U2OS cells were transfected with plasmids or shRNAs and subjected to NHEJ (**E**) and HR (**F**) assays, respectively. The ratio of GFP-positive cells in the Ku70-WT group was set as “1.0”. The data are presented as the means ± SDs from three independent experiments. *p* values determined by Student’s t test are shown. (**G**) Tumour growth curves after IR in xenograft mice. All data represent the means ± SDs. (**H**) Xenografts obtained from mice in the experimental groups. (**I**) The relative tumour doubling time in response to IR

To investigate the functional significance of IR-induced and nPORCN-dependent Ku70 *S-*palmitoylation, we generated shRNA-resistant Ku70 constructs (WT and 5 C/S) and expressed the constructs in stable Ku70 knockdown HT1080 cells (Supplemental Fig. 8D). Because palmitoylation might regulate the subcellular localization of proteins, we first detected the impact of cysteine mutations on Ku70 localization. As shown in Supplemental Fig. 8E, Ku70-5 C/S localization was not changed, indicating that palmitoylation might not affect the subcellular localization of Ku70. However, cells complemented with Ku70-5 C/S showed enhanced radiosensitivity (Fig. 8A). We also found that Ku70-5 C/S cells exhibited prolonged existence of IR-induced γ-H2AX (Fig. 8B **and C**), indicating that Ku70 cysteine mutations decreased the efficiency of DNA DSB repair. In addition, we observed the accumulation of γ-H2AX foci in Ku70-5 C/S cells during mitosis (Fig. 8D). We also focused on the NHEJ and HR systems to study whether Ku70 *S*-palmitoylation participates in DNA repair. As shown in Fig. 8E **and F**, similarly to the PORCN-knockdown and PORCN-ΔNLS constructs, the Ku70-5 C/S mutation resulted in defective NHEJ but not HR. These results indicate that nPORCN-mediated Ku70 *S-*palmitoylation is important for NHEJ.

To determine the effect of Ku70 *S-*palmitoylation on radiosensitivity in vivo, we transfected empty vector, Ku70-WT, or Ku70-5 C/S into Ku70-knockdown HT1080 cells. We used these cells to establish xenografts in nude mice. We found that the tumours in the Ku70-5 C/S group were much more sensitive to IR than those in the Ku70-WT group (Fig. 8G **and H**). We also found that the tumour doubling time significantly increased in the Ku70-5 C/S group after IR (Fig. 8I).

## Discussion

PORCN has been known for years as a multi-pass integral membrane protein on the endoplasmic reticulum that is responsible for the palmitoylation of Wnt proteins. However, PORCN has little known function beyond its role in the Wnt pathway. In our study, we prove the existence of a nuclear fraction of PORCN, termed nPORCN, and its independent role in the DDR. We found that nPORCN possesses *S-*palmitoylation activity, unlike ER membrane-bound PORCN in the cytosol, which exhibits *O*-acyltransferase activity. We identified nPORCN-interacting proteins and proved that Ku70 is a primary palmitoylation target in the DDR. We demonstrated that nPORCN-dependent palmitoylation is important for DNA-PKcs-Ku70-Ku80 complex formation and NHEJ. Together, these results highlight the functional significance of nPORCN-mediated *S-*palmitoylation in the DDR.

Potential mechanisms underlying the activation of nPORCN in the context of DNA damage include (1) an increase in nPORCN levels, resulting in increased PORCN translocation into the nucleus in response to IR; (2) no change in nPORCN levels but a change in nPORCN activity resulting from posttranslational modifications, such as phosphorylation, triggered by IR; or (3) a combination of an increase in nPORCN levels and an increase in nPORCN activation in response to IR. Our experimental findings demonstrate an augmented influx of PORCN into the nucleus (Fig. 4I). A recent study has shown that Ser332 of the PORCN isoform A (Ser 337 of PORCN isoform D, which has the NLS sequence) is responsible for engaging with the carbonyl oxygen of the PORCN inhibitor LGK974 [39]. Consequently, it is plausible that phosphorylation of PORCN Ser337 may be essential for the activity of PORCN. Further investigation of upstream kinases required for nPORCN activation in the DDR is warranted.

PORCN-null cells did not exhibit impaired ATM activation in response to IR (Supplemental Fig. 3A and 3B), indicating that PORCN is not required for IR-induced ATM activation. Therefore, the regulatory effect of ATM on HR is unaffected in the U2OS-DR reporter system upon suppression of PORCN. Nevertheless, we observed compromised formation of the DNA-PKcs/Ku70/Ku80 complex in PORCN-KO cells. Consequently, PORCN inhibition results in diminished NHEJ efficiency. Further evidence reveals that nPORCN-dependent, IR-induced Ku70 palmitoylation is critical for NHEJ. Although our experiments did not find Ku80 and DNA-PKcs to be direct targets of PORCN, it is likely that there are other DDR proteins that are palmitoylated in the process.

Gao et al. reported that PORCN catalyses the palmitoylation of Wnt3a at Ser209 but not Cys77, indicating that PORCN is an *O*-acyltransferase [38]. In our study, we prove a new function of nPORCN with *S-*acyltransferase activity, indicating that PORCN possesses dual palmitoylation activities. Although the catalytic mechanism of nPORCN is still unclear, it is likely that the microenvironmental differences between the nucleus and cytosol might trigger differential enzymatic activities.

Despite the limited insights obtained from the structural simulation of Ku70 palmitoylation, it is plausible to suggest that Ku70 palmitoylation plays a crucial role in the activation-associated phosphorylation of the DNA-PK complex. This hypothesis is substantiated by the observed disruption of DNA-PKcs autophosphorylation in PORCN NLS-KO cells, where Ku70 palmitoylation is inhibited (Supplemental Fig. 4D). The spatial conformation of proteins, as well as their intermolecular interactions and signalling, can be influenced by posttranslational modifications. In our investigation, we observed that following palmitoylation of Ku70, it exhibits increased affinity for the Ku80 and DNAPK complexes, potentially attributable to the altered spatial arrangement of Ku70 resulting from palmitoylation. Consequently, this modification facilitates its association with downstream complexes.

As DNA damaging agents (such as radiotherapy and many chemotherapeutic compounds) remain mainstream cancer therapies, understanding how cellular sensitivity to these therapies is controlled by various signalling pathways should provide important leads for molecular radio- and chemosensitization. Our results show that PORCN knockdown leads to hypersensitivity to IR, indicating that PORCN could serve as a target for radio- and chemosensitization. Indeed, LGK974, a small-molecule inhibitor of PORCN, has been shown to be a radiosensitizer [40]. Since many PORCN inhibitors are in clinical trials for solid tumours with active Wnt signalling and radiotherapy is a major treatment option for such tumours, it is likely that PORCN inhibitors would exert beneficial effects in these patients when used concurrently with radiotherapy.

**Fig. 9:**
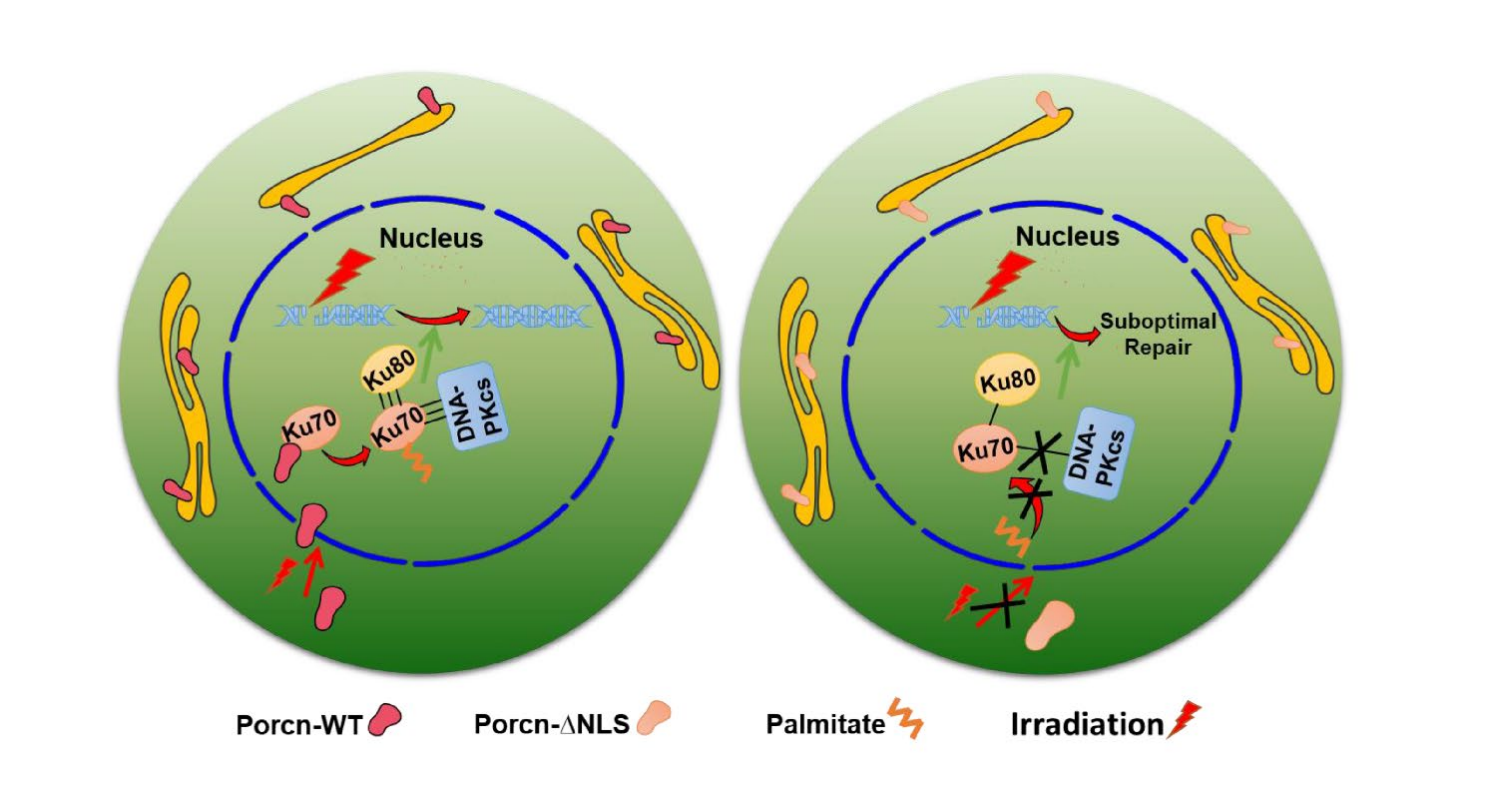
A schematic model of nPORCN-dependent Ku70 S-palmitoylation during the DNA damage response

In conclusion, we demonstrate that a fraction of PORCN exists in the nucleus and is activated in response to DNA damage (Fig. 9). We also reveal that nPORCN is needed for Ku70 *S-*palmitoylation and that this process is important for the formation of the DNA-PKcs, Ku70 and Ku80 complex as well as the activation of NHEJ. These findings provide an in-depth understanding of the regulatory mechanism of the DDR.

## Electronic supplementary material

Below is the link to the electronic supplementary material.

Supplementary Material 1

Supplementary Material 2

## Data Availability

No datasets were generated or analysed during the current study.

## Abbreviations

DDR: DNA damage response
PTMs: Post-translational modifications
ABE: Acyl-biotin exchange
PORCN: Porcupine
NHEJ: Nonhomologous end joining
IR: Ionizing radiation
nPORCN: Nuclear PORCN
UV: Ultraviolet radiation
DSBs: DNA double-strand breaks
HR: Homologous recombination
DNA-PKcs: DNA-dependent protein kinase catalytic subunit
PATs: Palmitoyl acyltransferases
HHAT: Hedgehog acyltransferase
GOAT: Ghrelin acyltransferase
MBOAT: Membrane-bound O-acyltransferase
Fzd: Frizzled
FA: Fanconi anemia
NLS: Nuclear localization sequence
KO: Knockout
co-IP: Coimmunoprecipitation
HAM: Hydroxylamine
Cys: Cysteine
LB: Lysis buffer
Biotin-BMCC: Biotinylated Maleimide Cyclohexane Carboxylate
