# Supplementary material for: Nuclear porcupine mediates XRCC6/Ku70 S-palmitoylation in the DNA damage response
Source: Exp Hematol Oncol. 2024 Nov 4;13:109. doi: 10.1186/s40164-024-00572-w (PMC11536954; doi:10.1186/s40164-024-00572-w)
Supplement: Supplementary file 1 — Supplementary Material 1 [file 40164_2024_572_MOESM1_ESM.pdf]

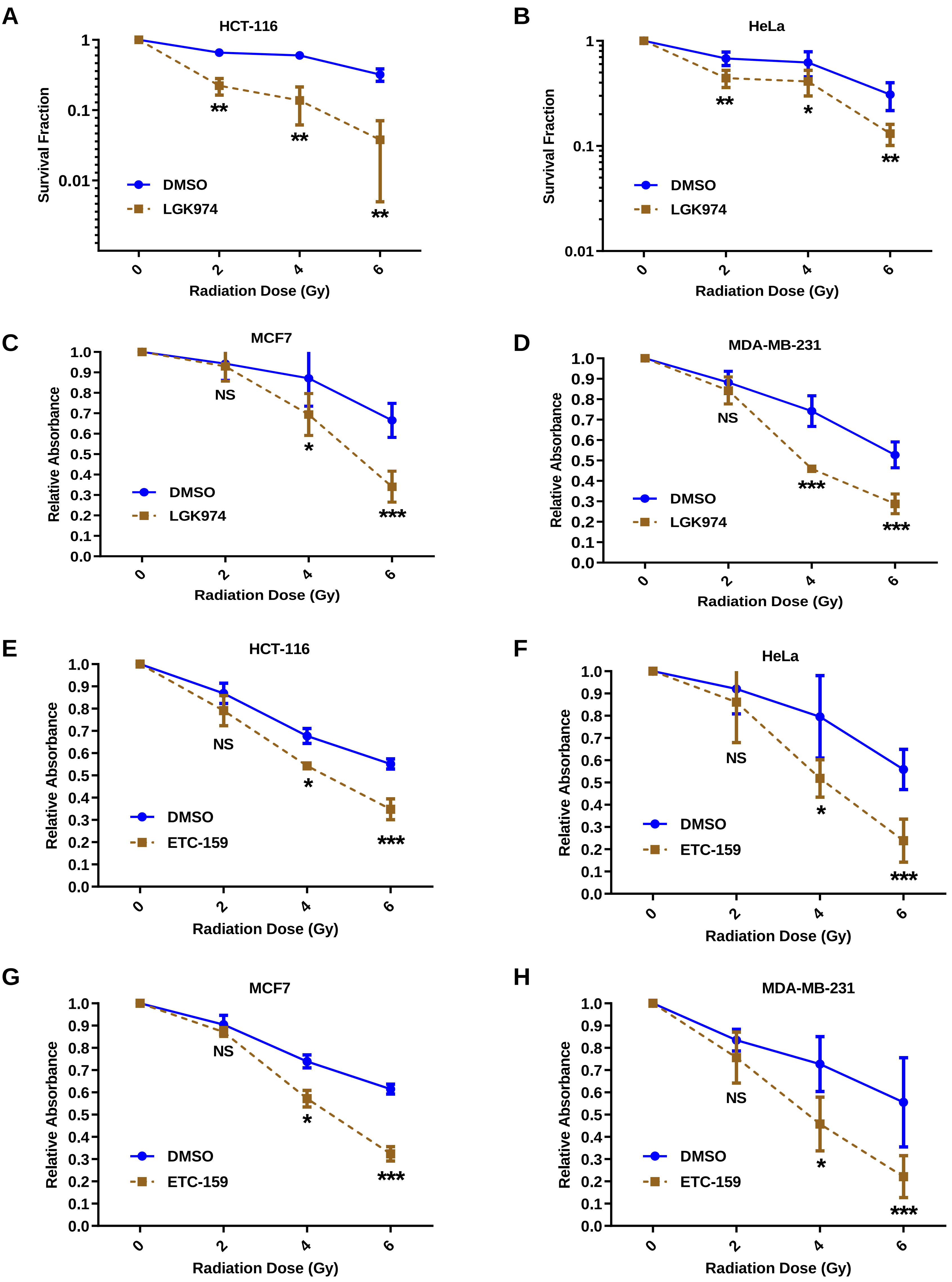

Supplemental Figure 1

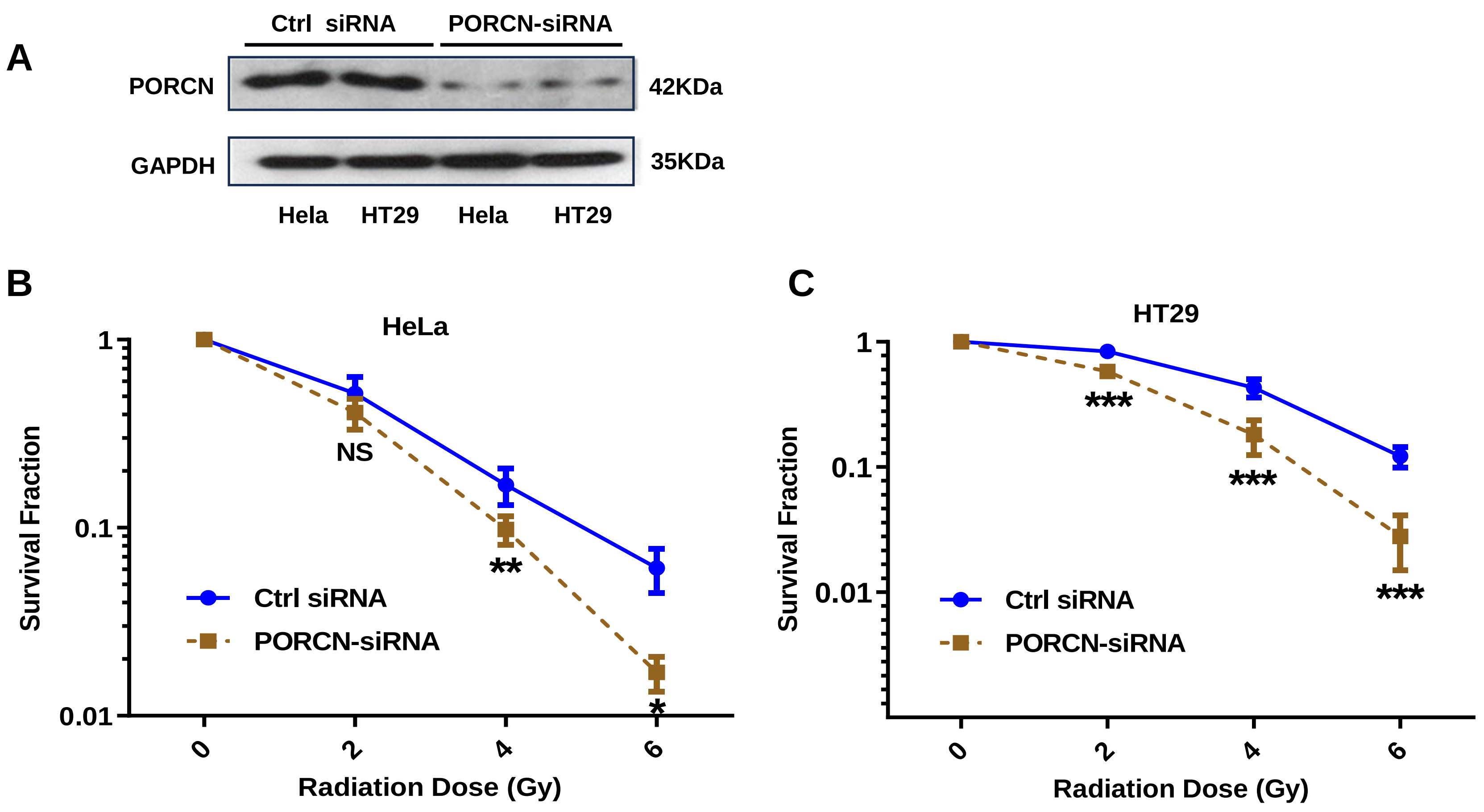

Supplemental Figure 2

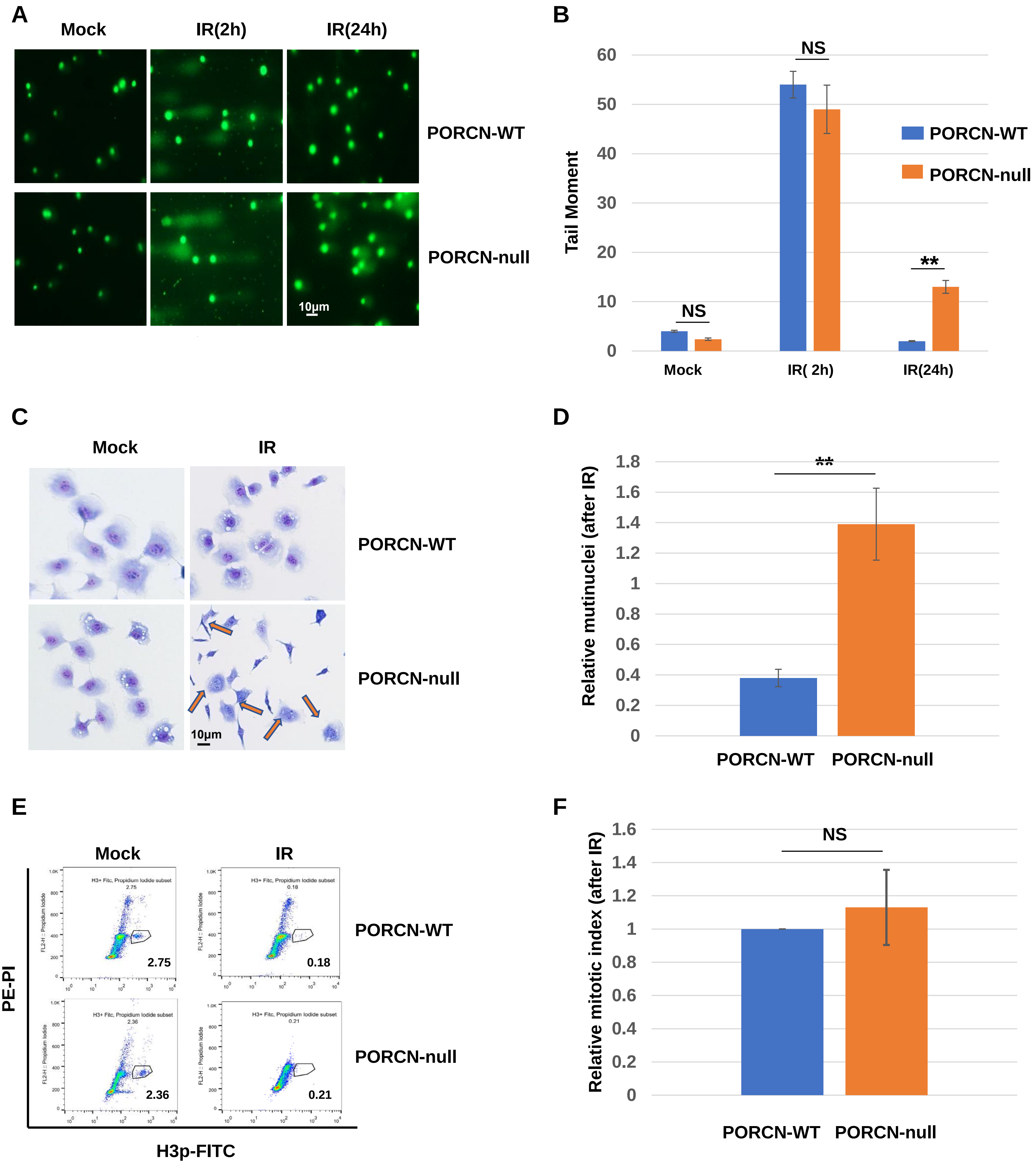

Supplemental Figure 3

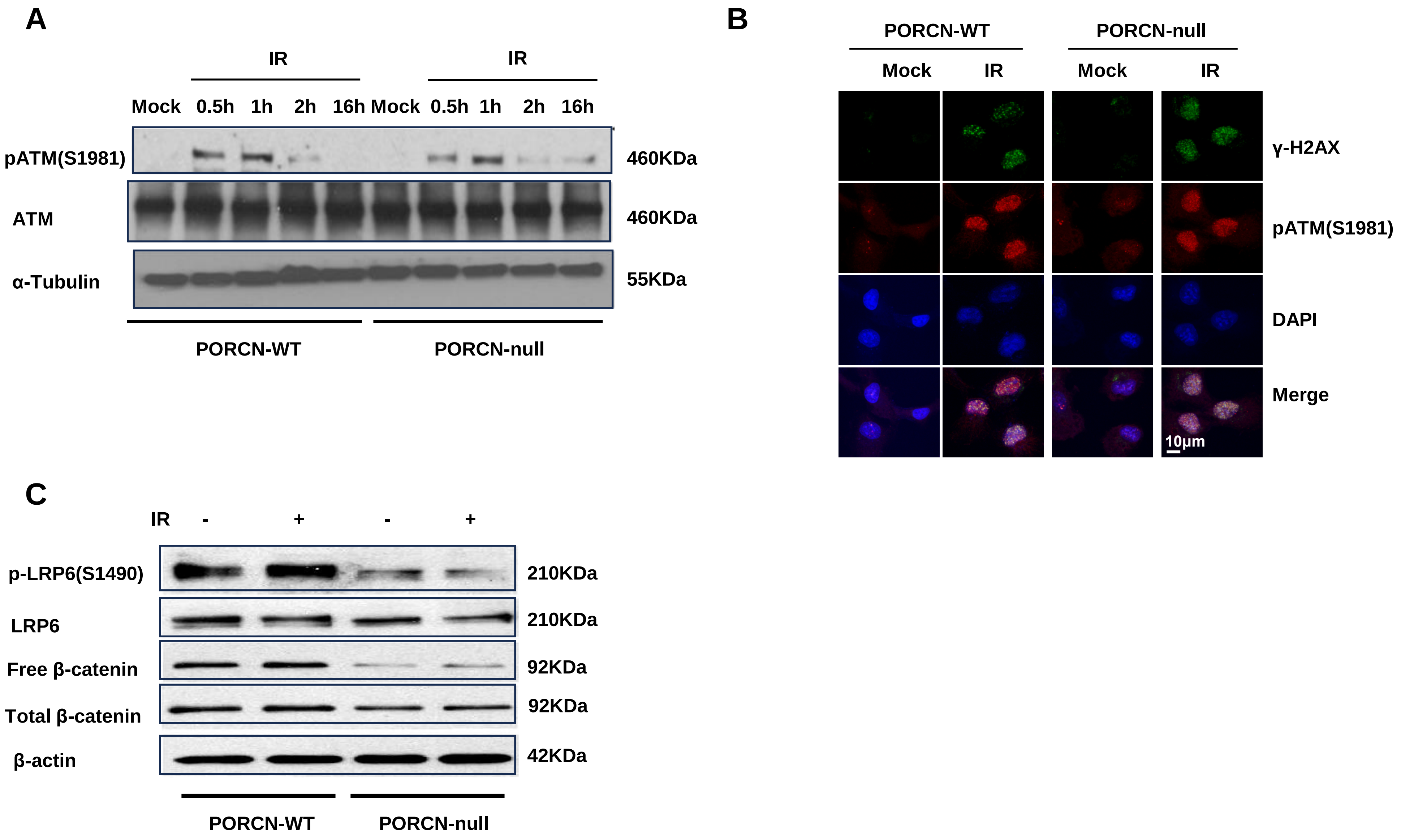

Supplemental Figure 4

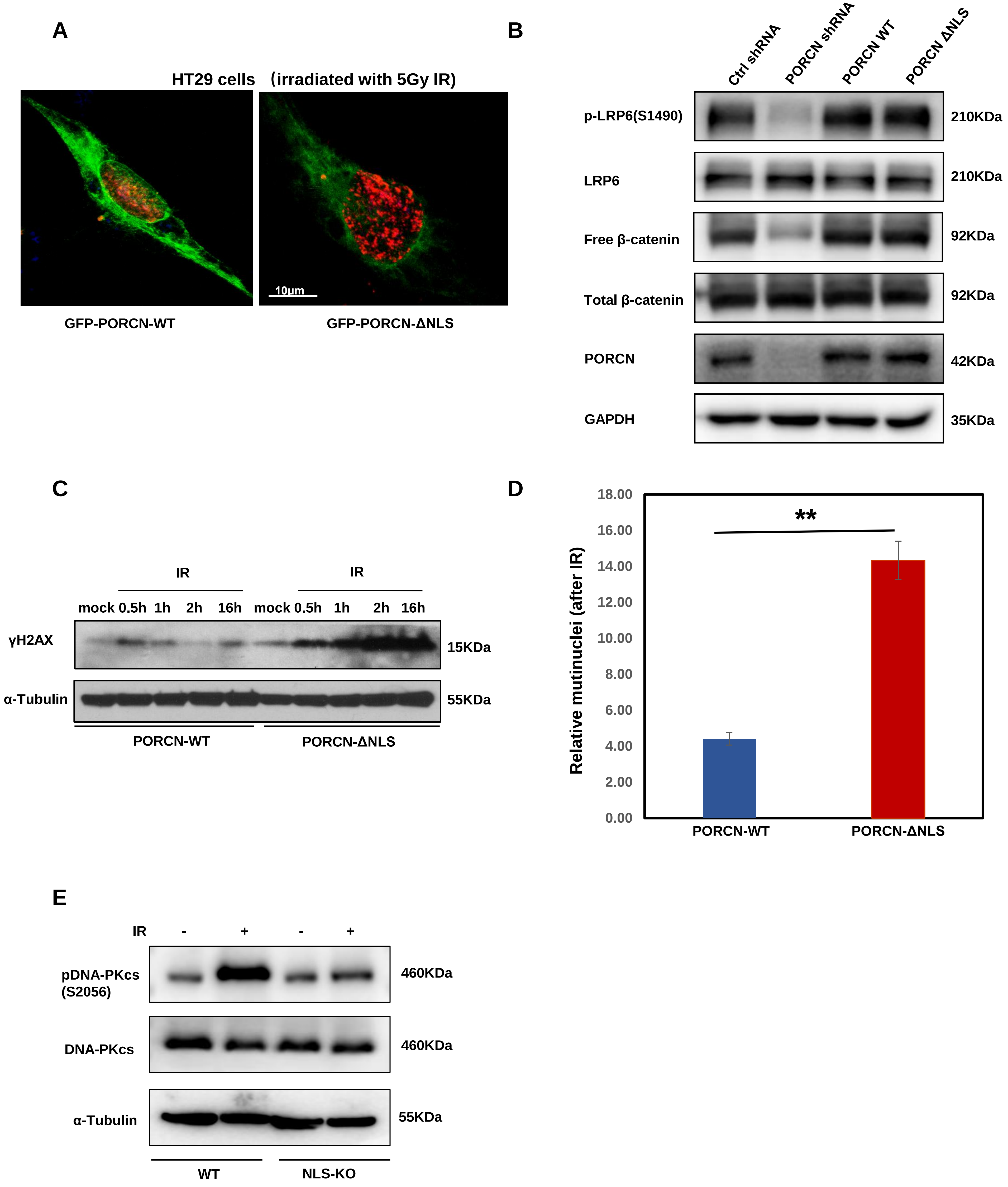

Supplemental Figure 5

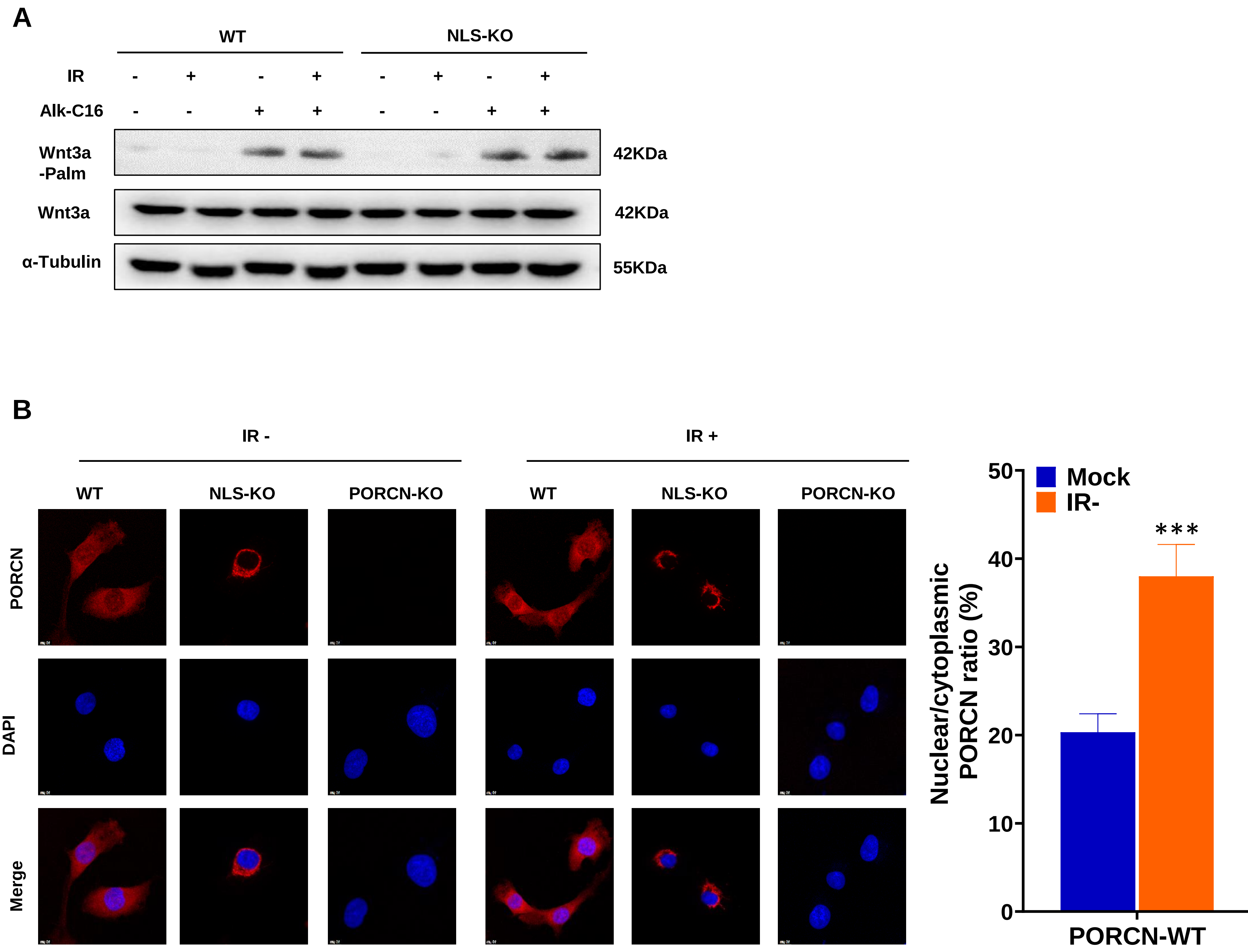

Supplemental Figure 6

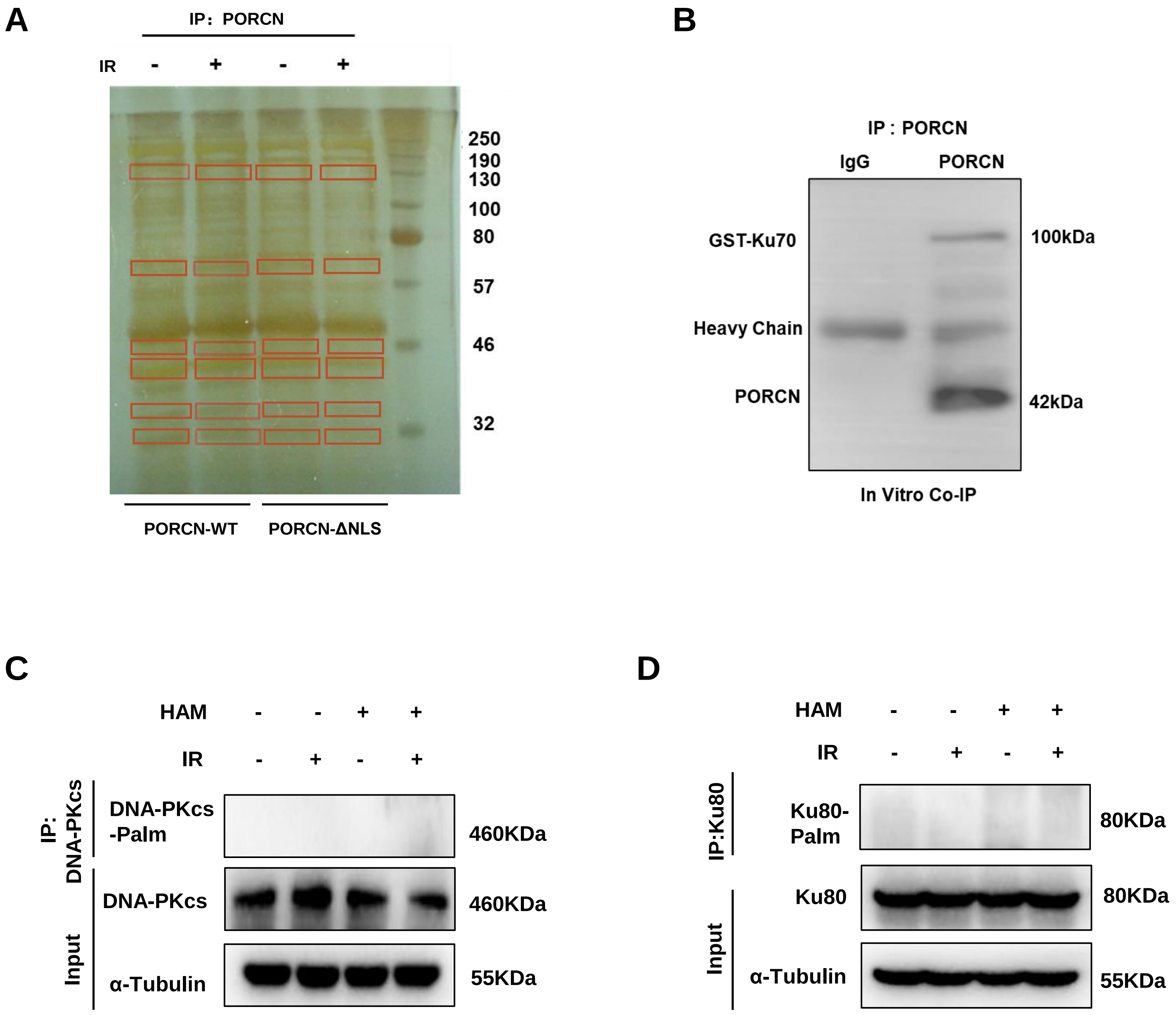

Supplemental Figure 7

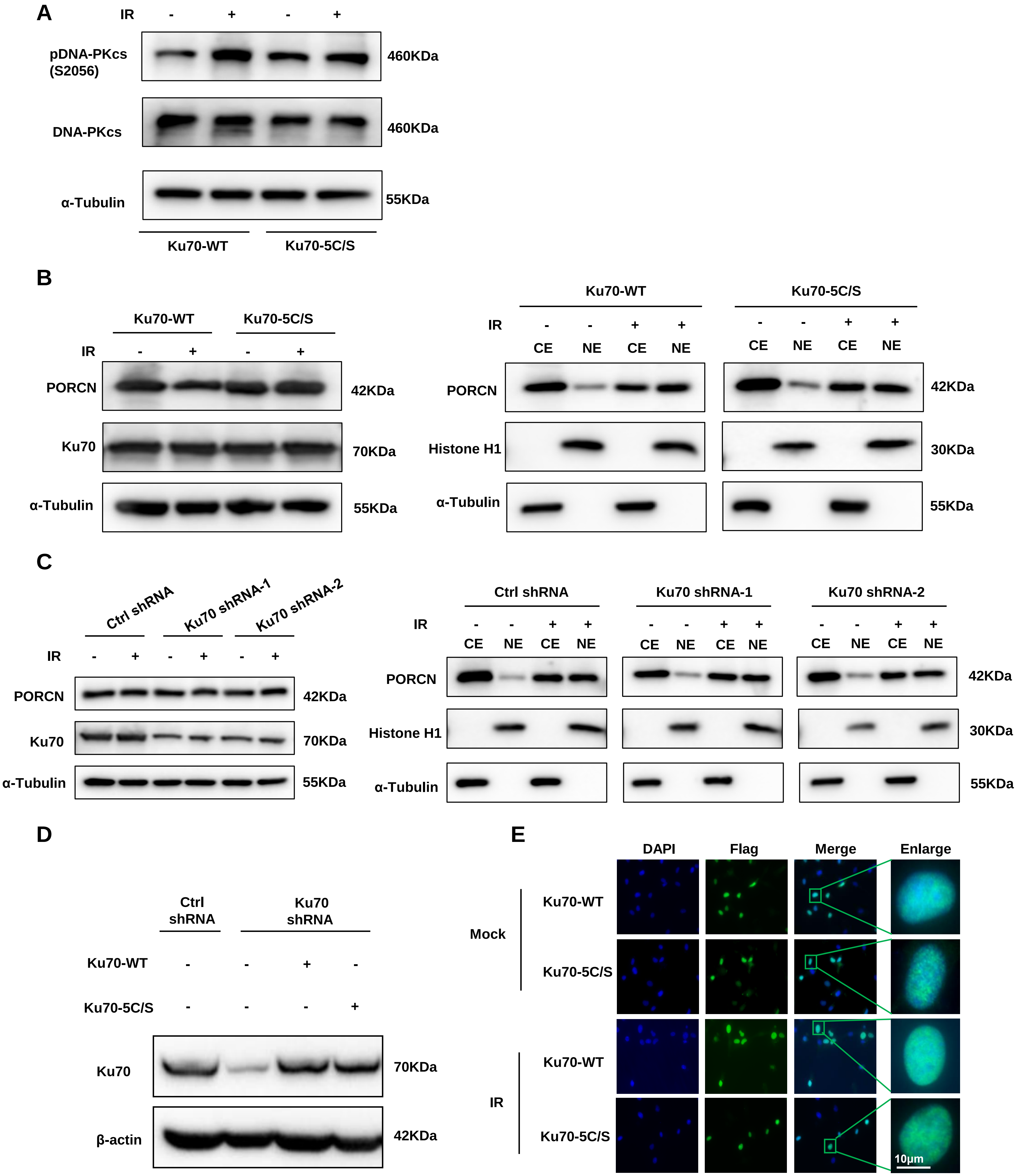

Supplemental Figure 8

Supplemental Figure Legends

Supplemental Figure 1. (A-B) Surviving fractions were measured by the colony formation assay in HCT-116(A) and HeLa(B) cells in the presence of DMSO or LGK974 (1nM for 24 h). p values from Student's t test are shown. (C-D) MCF-7(C) and MDA-MB-231(D) cells treated with DMSO and LGK974 (1nM for 24 h) were irradiated and assessed via the MTT assay. p values from Student's t test are shown. (E-H) HCT-116 (E), HeLa (F), MCF-7(G) and MDA-MB-231(H) cells treated with DMSO and ETC-159 (2.9nM for 24 h) were irradiated and assessed via the MTT assay. The data are presented as the means ± SDs from three independent experiments, and p values from Student's t test are shown.

Supplemental Figure 2. (A) Expression of PORCN measured by Western blot analysis after transfection with control siRNA and PORCN siRNA in Hela and HT29 cells. (B-C) Radiosensitivity was measured by the colony formation assay in HeLa(F) and HT29(G) cells expressing control siRNA or PORCN siRNA in the presence of DMSO or LGK974 (1nM for 24 h). The data are presented as the means ± SDs from three independent experiments, and p values from Student's t test are shown.

Supplemental Figure 3. (A) Control or PORCN-null HT1080 cells were irradiated and then subjected to the comet assay at indicated timepoint. Scale bar: 10 μm. (B) Quantification of the tail moment in Control or PORCN-null HT1080 cells. The data are presented as the means ± SDs from three independent experiments. At least 50 cells were analyzed. p values from Student's t test are shown. (C) Multi-nucleation and micro-nucleation were measured by Giemsa staining and quantified in (D). The data are presented as the means ± SDs from three independent experiments. At least 50 cells were analyzed. p values from Student's t test are shown. (E-F) Control or PORCN-null HT1080 cells were treated with mock or IR before they were stained with a

flow cytometry–based anti-phospho-histon H3 Ser10 staining to determine the mitotic index. Representative photos are shown in (E) and quantification data in (F). The data are presented as the means ± SDs from three independent experiments. At least 50 cells were analyzed. p values from Student's t test are shown.

Supplemental Figure 4. (A) Whole cell extracts from indicated HT1080 cells treated with 5Gy IR were harvested and subjected to SDS-PAGE and immunoblotted with indicated antibodies. (B) PORCN-WT or PORCN-null HT1080 cells were subjected to mock treatment or IR. At 16 hours after IR, the cells were fixed, stained with anti-pATM(S1981) and γ-H2AX antibodies and DAPI, and subjected to immunofluorescence microscopy. Scale bar: 10 μm. (C) Whole cell extracts from indicated HT1080 cells treated with 5Gy of IR were harvested and subjected to SDS-PAGE and immunoblotting with indicated antibodies.

Supplemental Figure 5. (A) Confocal microscopic images of localization patterns of GFP-tagged PORCN-WT (left) or PORCN-ΔNLS (right) in irradiated HT29 cells. Green represents GFP and red represents γ-H2AX. Scale bar:10 μm. (B) Whole-cell extracts from the indicated HT1080 cells treated with 5 Gy of IR were harvested and subjected to SDS-PAGE and immunoblotting with the indicated antibodies. (C) The indicated HT29 cells were mock-treated or irradiated with 5 Gy and released at the indicated time points. Total cell lysates were harvested and subjected to Western blot analysis using the indicated antibodies. (D) Rosettes formation of indicated HT1080 cells (multi-nucleation and micro-nucleation) were quantified. The data are presented as the means ± SDs from three independent experiments. At least 50 cells were analyzed. p values from Student's t test are shown. (E) Whole cell extracts from indicated HT1080 cells treated with 5Gy IR were harvested and subjected to SDS-PAGE and immunoblotted with indicated antibodies.

Supplemental Figure 6. (A) Wnt3a was immunoprecipitated from in HT1080 PORCN-WT and PORCN NLS-KO cells, and a click-chemistry assay was performed. Western blot analysis was carried out using the indicated antibodies. (B) Confocal microscopic images of HT1080 PORCN-WT, PORCN-KO, and NLS-KO cells were treated with or without IR (5 Gy). After 2 h, the cells were fixed, stained with anti-PORCN antibodies and DAPI, and subjected to immunofluorescence microscopy. Scale bar: 10 μm. The nuclear/cytoplasmic PORCN ratio was quantified. The data are presented as the means ± SDs from three independent experiments.

Supplemental Figure 7. (A) SDS-PAGE separation of PORCN WT or PORCN-ΔNLS immunoprecipitates. The gel was visualized using silver staining. (B) The GST-tagged Ku70 protein was incubated with recombinant PORCN at 4°C for 2 h in HT1080 cells, and PORCN was immunoprecipitated with Protein A/G beads bound to the PORCN antibody in vitro and analyzed by Western blotting using PORCN and GST antibody. (C-D) HT1080 cells were subjected to mock or IR. (C) DNA-PKcs or (D) Ku80 was immunoprecipitated and conducted for the ABE assay in the presence of HAM. The palmitoylated proteins were immunoblotted with the Streptavidin-HRP antibody.

Supplemental Figure 8. (A) Whole cell extracts from HT1080 cells transfected with indicated plasmids were treated with or without IR (5 Gy). After 2 h, the cells were subjected to SDS-PAGE and immunoblotted with indicated antibodies. (B-C) Cytoplasmic and nuclear extracts from HT1080 cells transfected with indicated plasmids were treated with or without IR (5 Gy). After 2 h, the cells were subjected to SDS-PAGE and immunoblotted with anti-PORCN, anti-histone H1, and anti-α-tubulin antibodies. (D) Expression of shRNA-resistant Ku70 constructs (WT and 5C/S) in stable Ku70 knockdown HT1080 cells. (E) Flag-Ku70-WT or Flag-Ku70-5C/S HT1080 cells were treated with or without IR. At 2 hours after IR, cells were fixed and subjected to immunofluorescence staining with the anti-flag antibody and DAPI. Shown are

confocal microscopic images of mock-treated or irradiated cells. Scale bar: 10μm.
