# Supplementary material for: Nuclear porcupine mediates XRCC6/Ku70 S-palmitoylation in the DNA damage response
Source: Exp Hematol Oncol. 2024 Nov 4;13:109. doi: 10.1186/s40164-024-00572-w (PMC11536954; doi:10.1186/s40164-024-00572-w)

Uncropped blot of Fig.4F

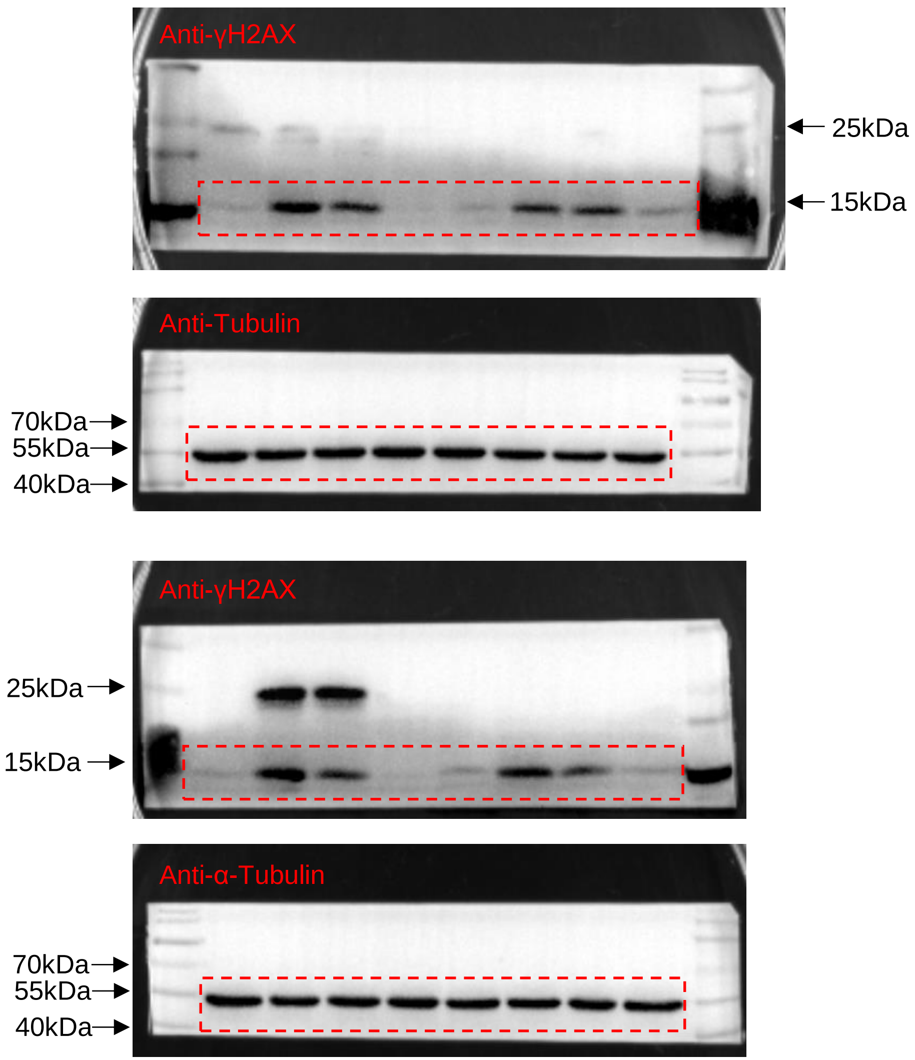

Uncropped blot of Fig.4H

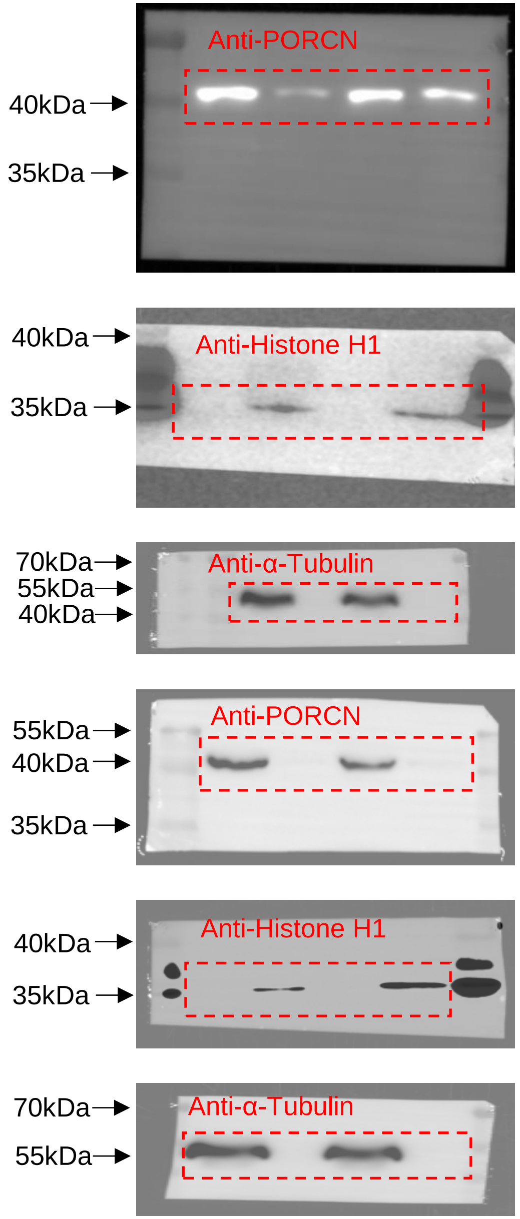

Uncropped blot of Fig.5B

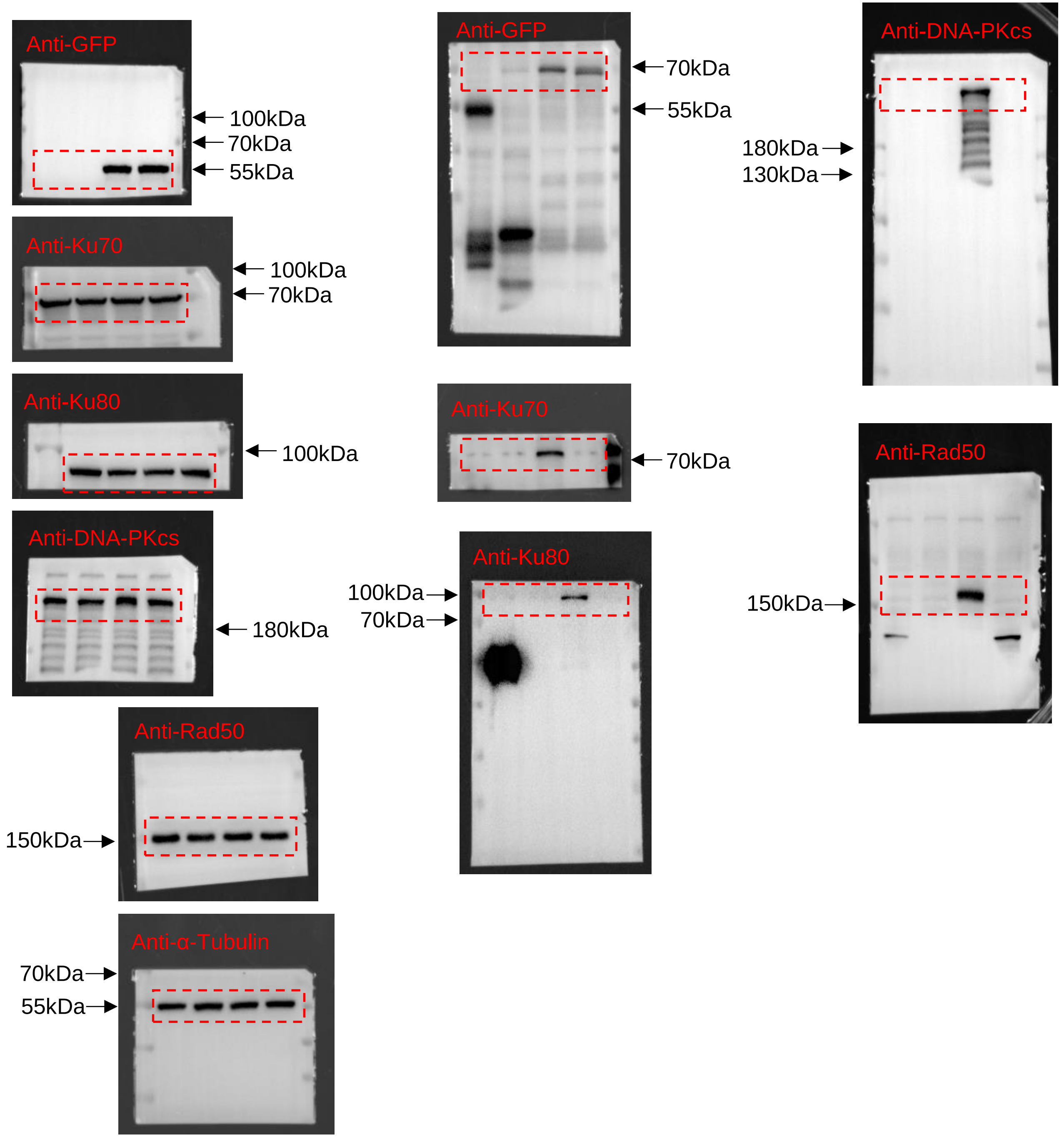

Uncropped blot of Fig.5C

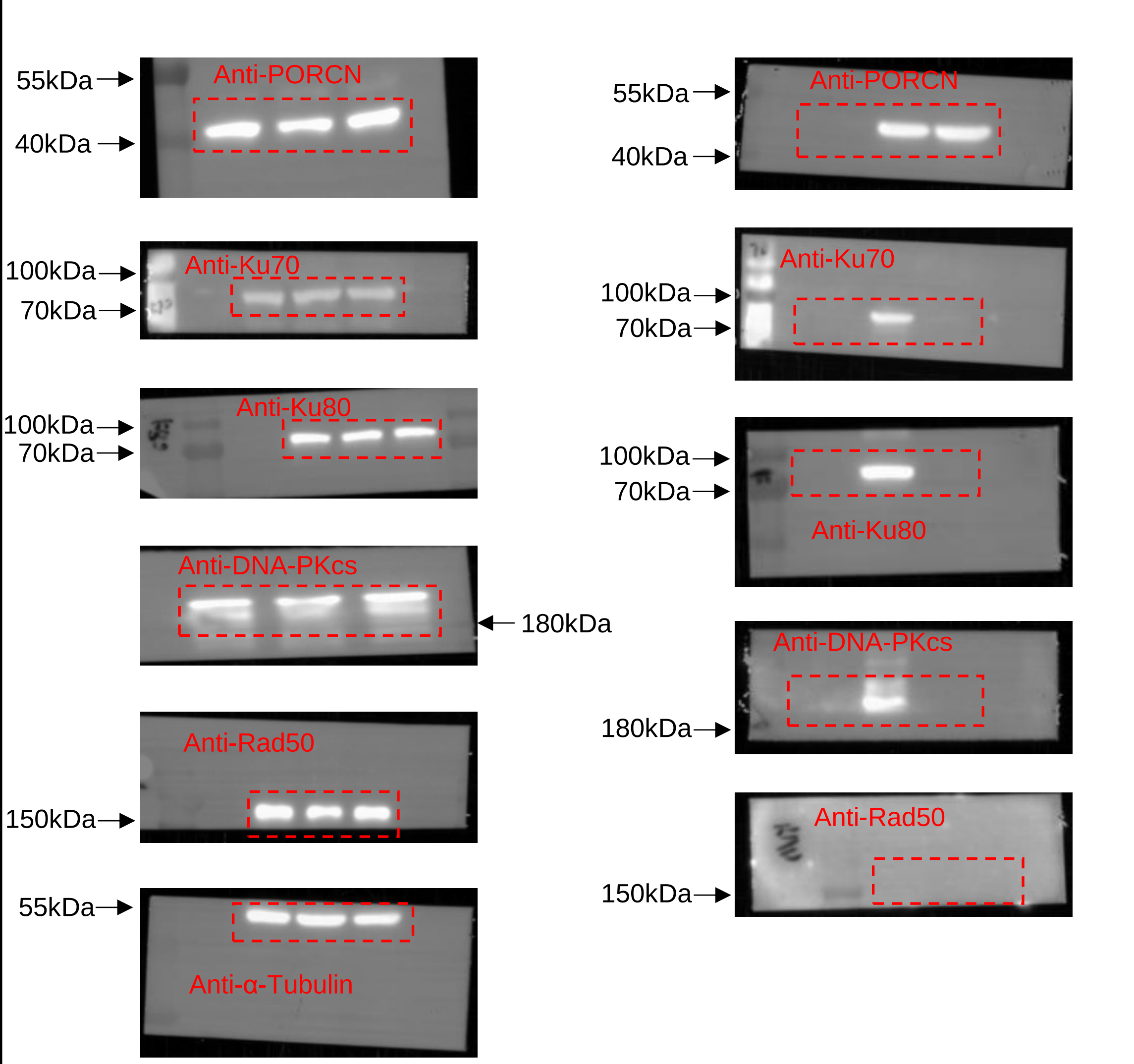

Uncropped blot of Fig.5D

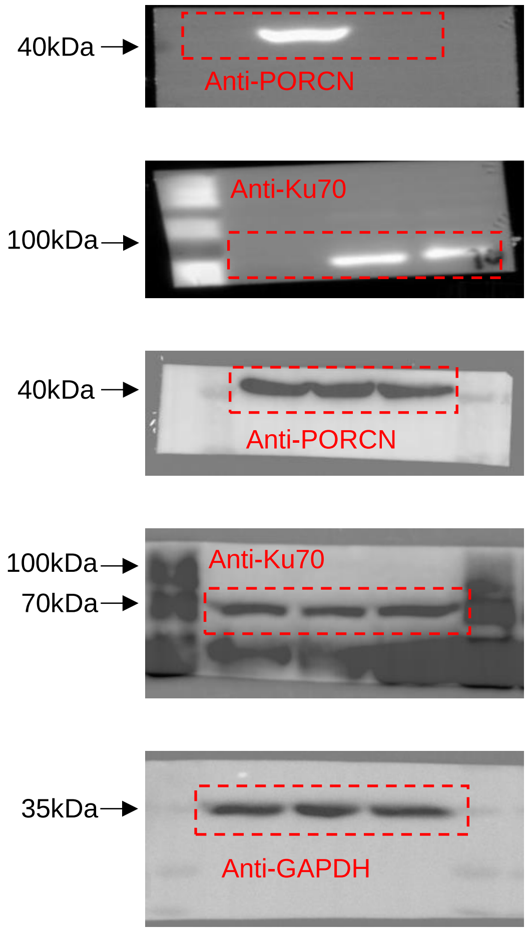

Uncropped blot of Fig.5E

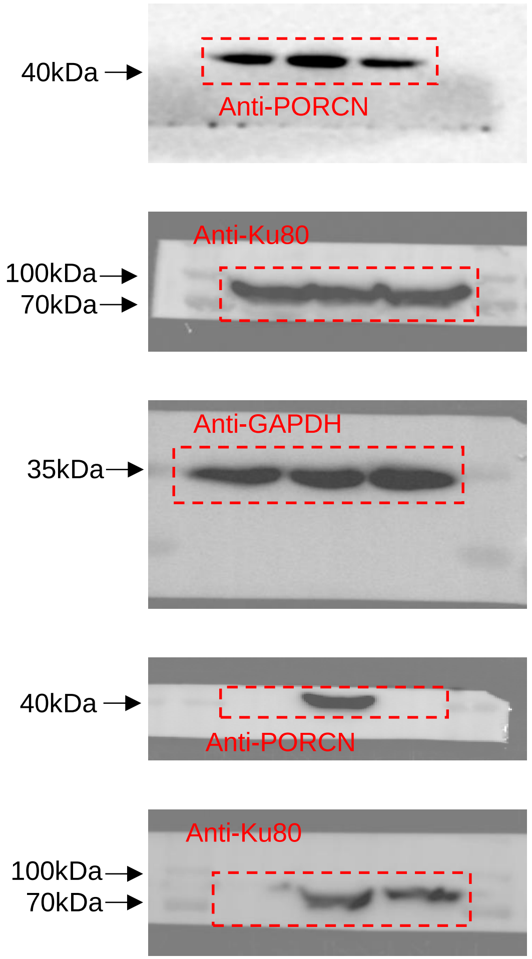

Uncropped blot of Fig.5F

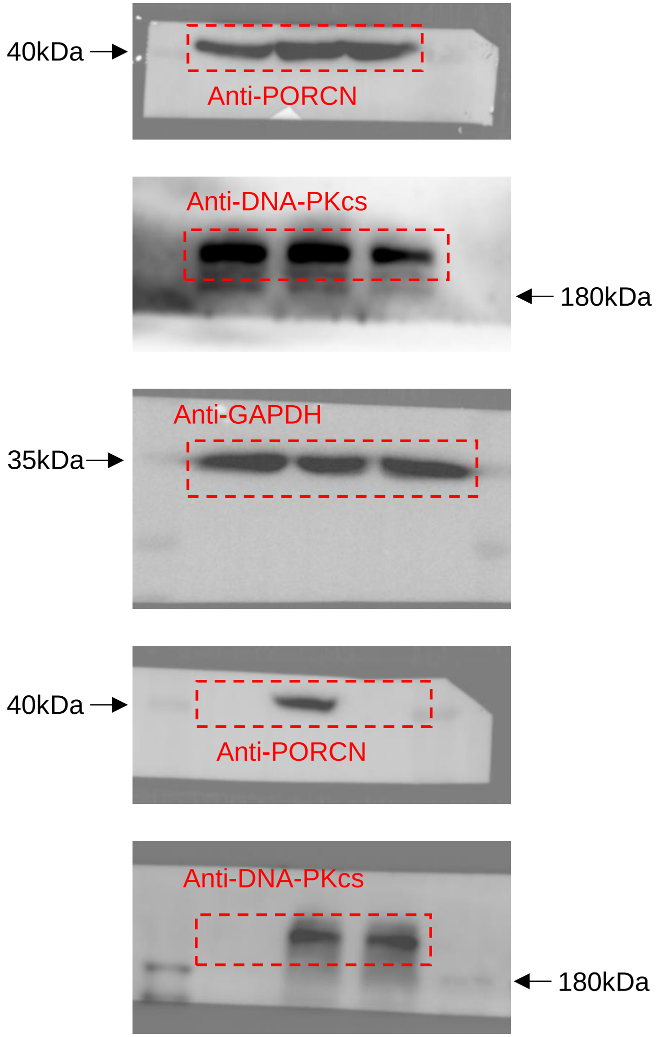

Uncropped blot of Fig.6B

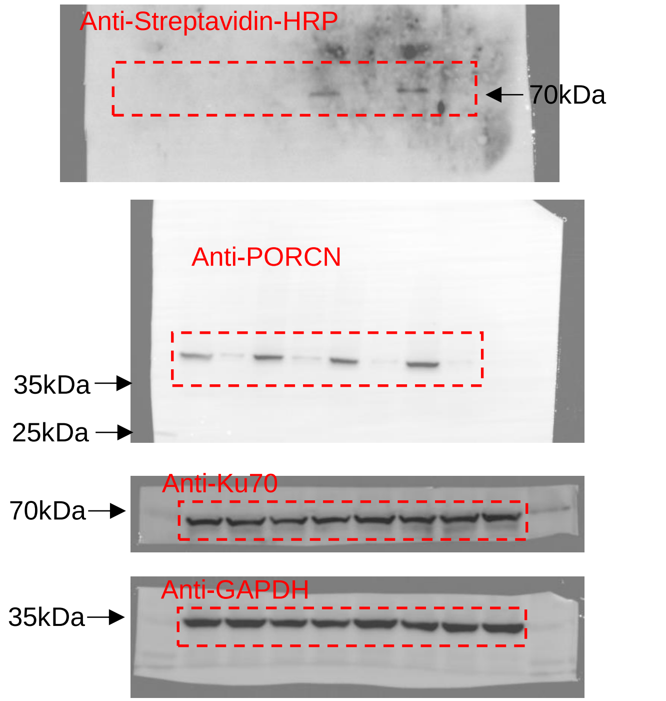

Uncropped blot of Fig.6C

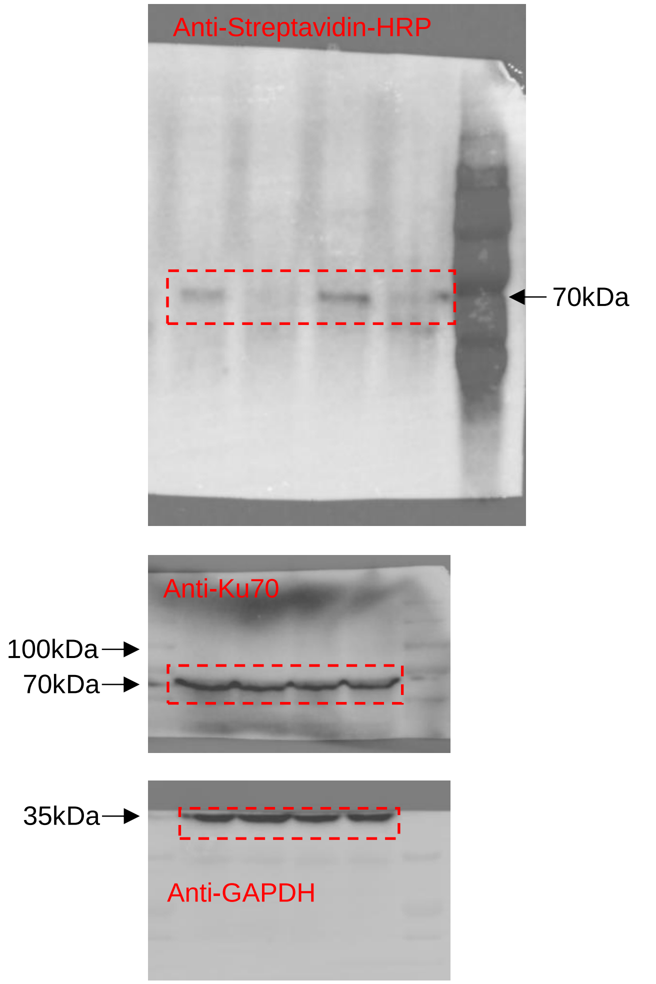

Uncropped blot of Fig.6E

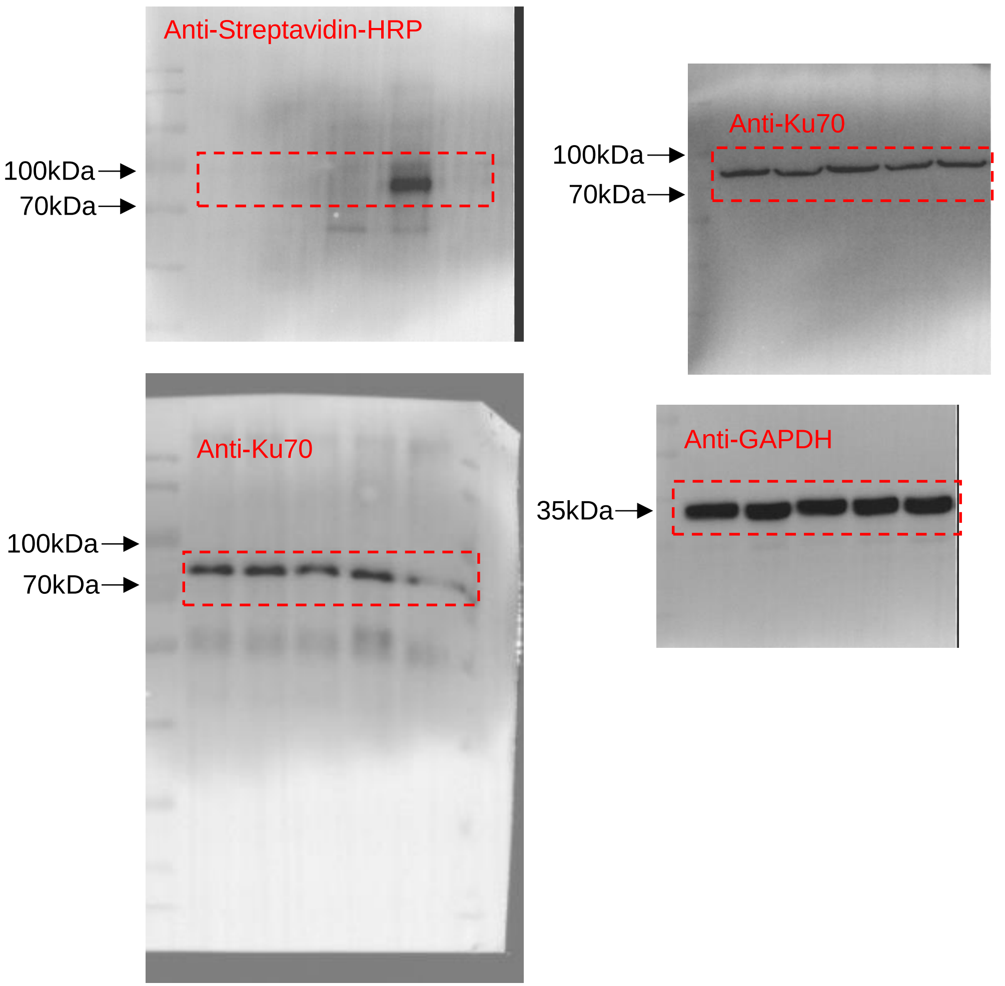

Uncropped blot of Fig.6F

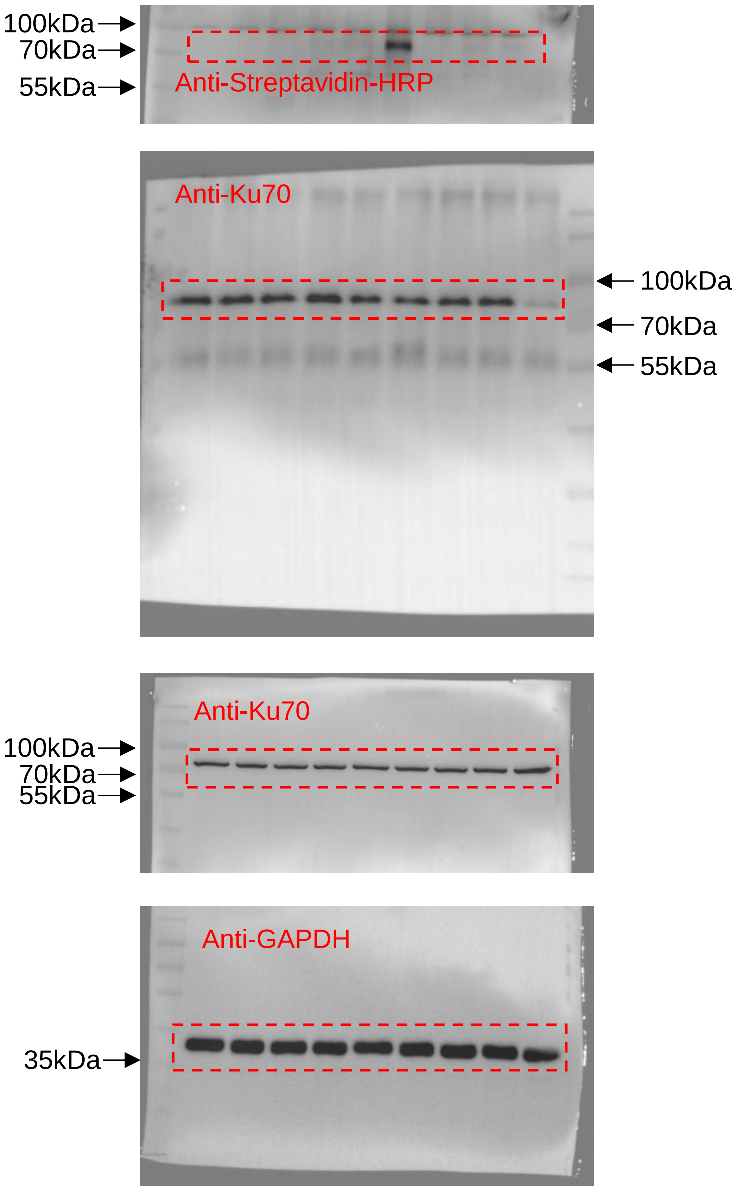

Uncropped blot of Fig.6G

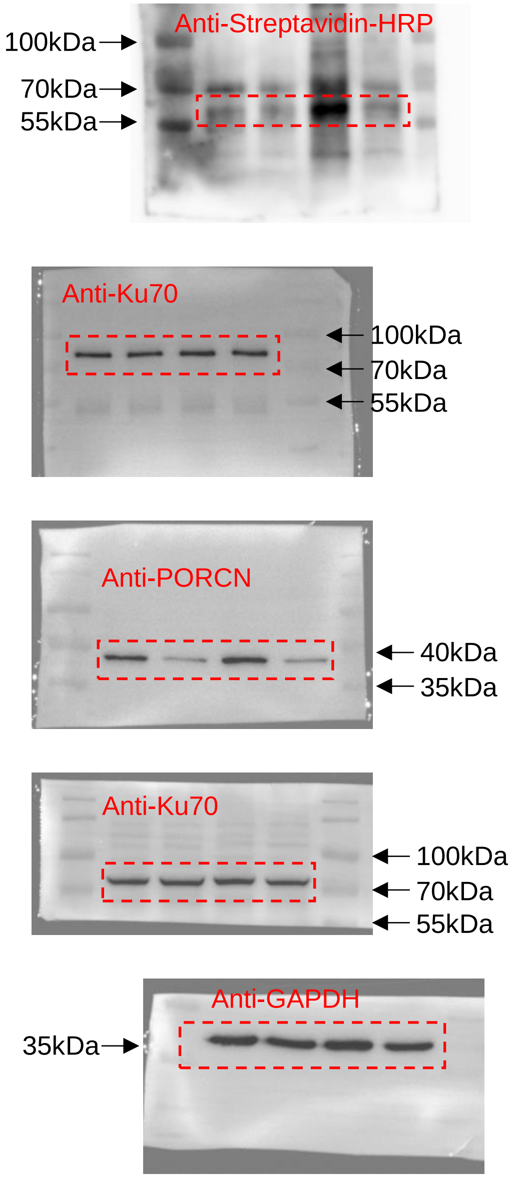

Uncropped blot of Fig.6H

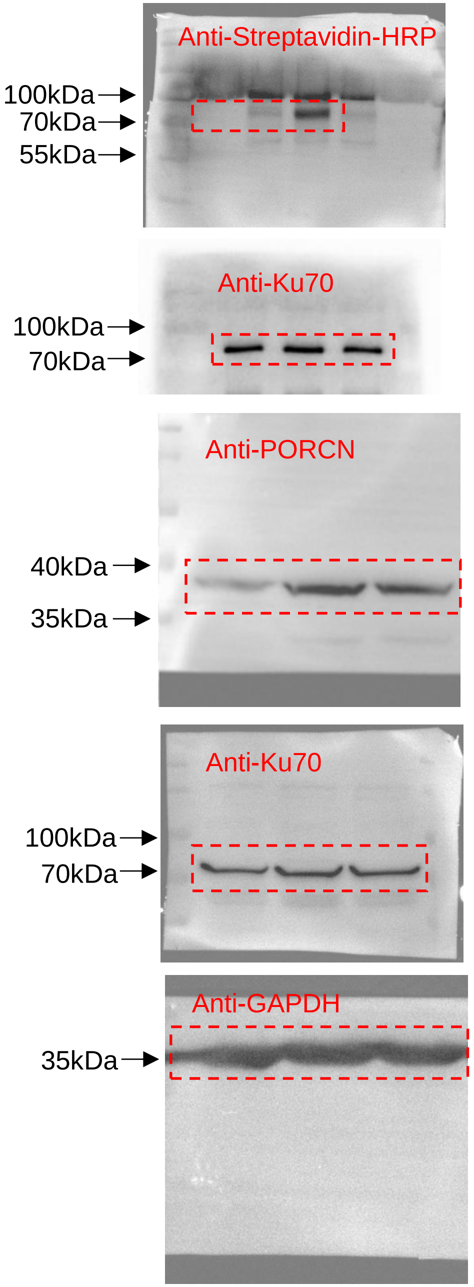

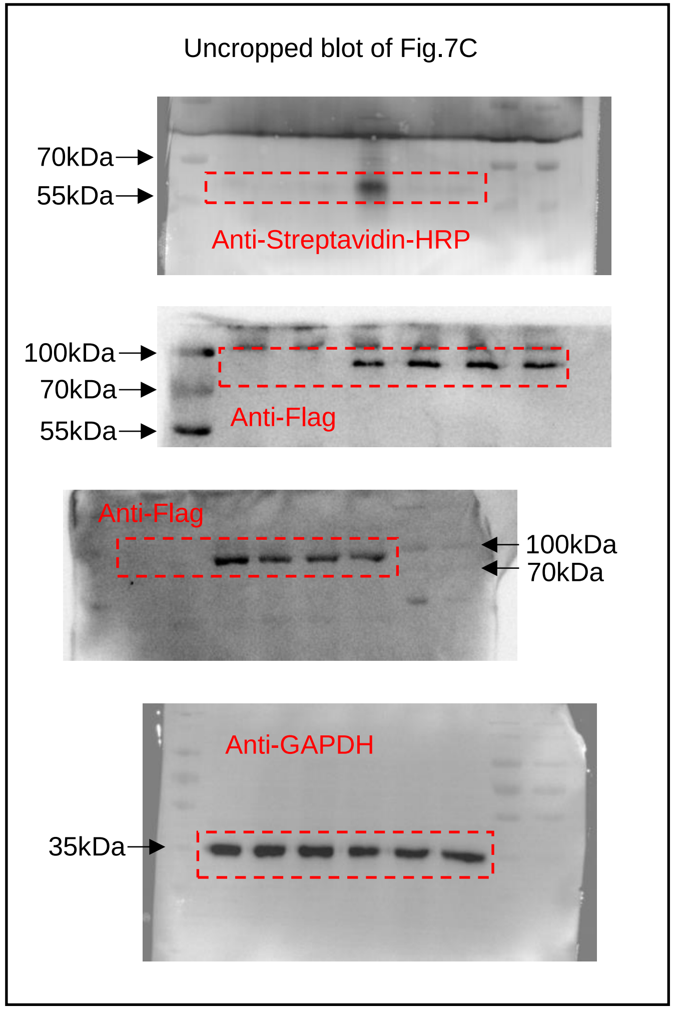
Uncropped blot of Fig.7C
70kDa
55kDa
Anti-Streptavidin-HRP
100kDa
70kDa
55kDa
Anti-Flag
Anti-Flag
100kDa
70kDa
Anti-GAPDH
35kDa

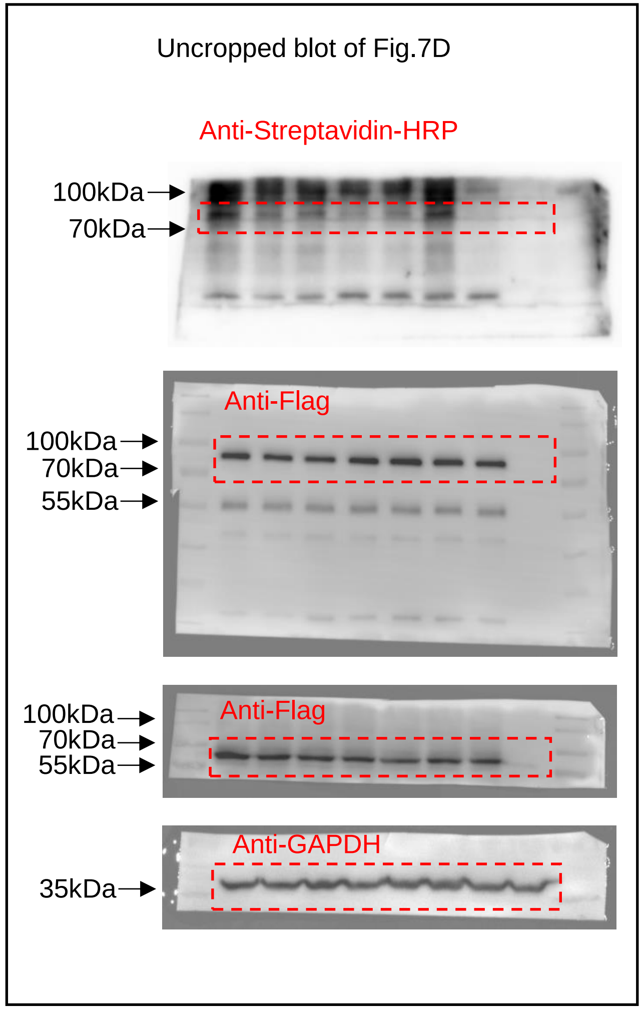
Uncropped blot of Fig.7D
Anti-Streptavidin-HRP
100kDa
70kDa
Anti-Flag
100kDa
70kDa
55kDa
Anti-Flag
100kDa
70kDa
55kDa
Anti-GAPDH
35kDa

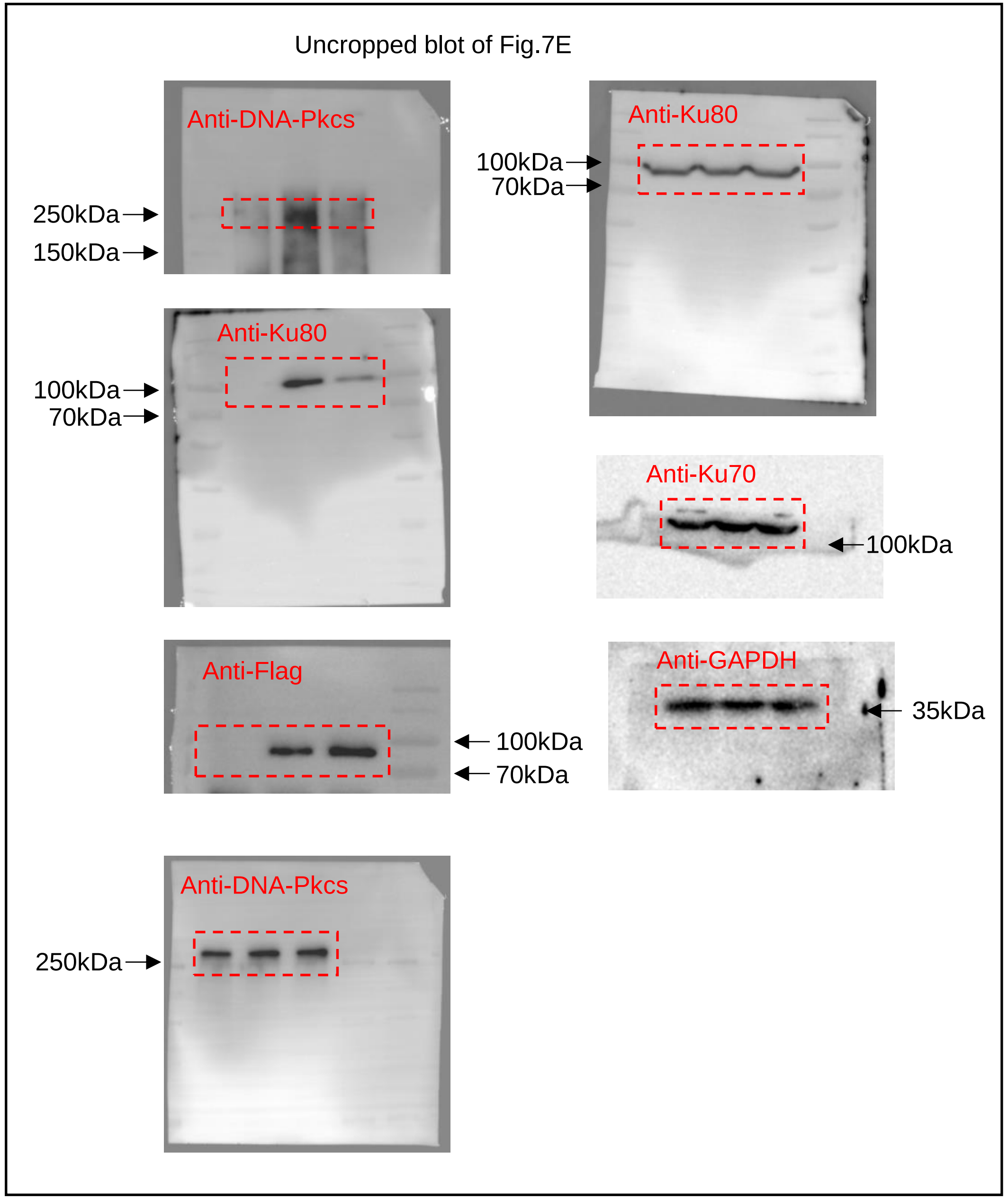
Uncropped blot of Fig.7E
Anti-DNA-Pkcs
250kDa
150kDa
Anti-Ku80
100kDa
70kDa
Anti-Ku80
100kDa
70kDa
Anti-Ku70
100kDa
Anti-Flag
100kDa
70kDa
Anti-GAPDH
35kDa
Anti-DNA-Pkcs
250kDa

Uncropped blot of Fig.7F

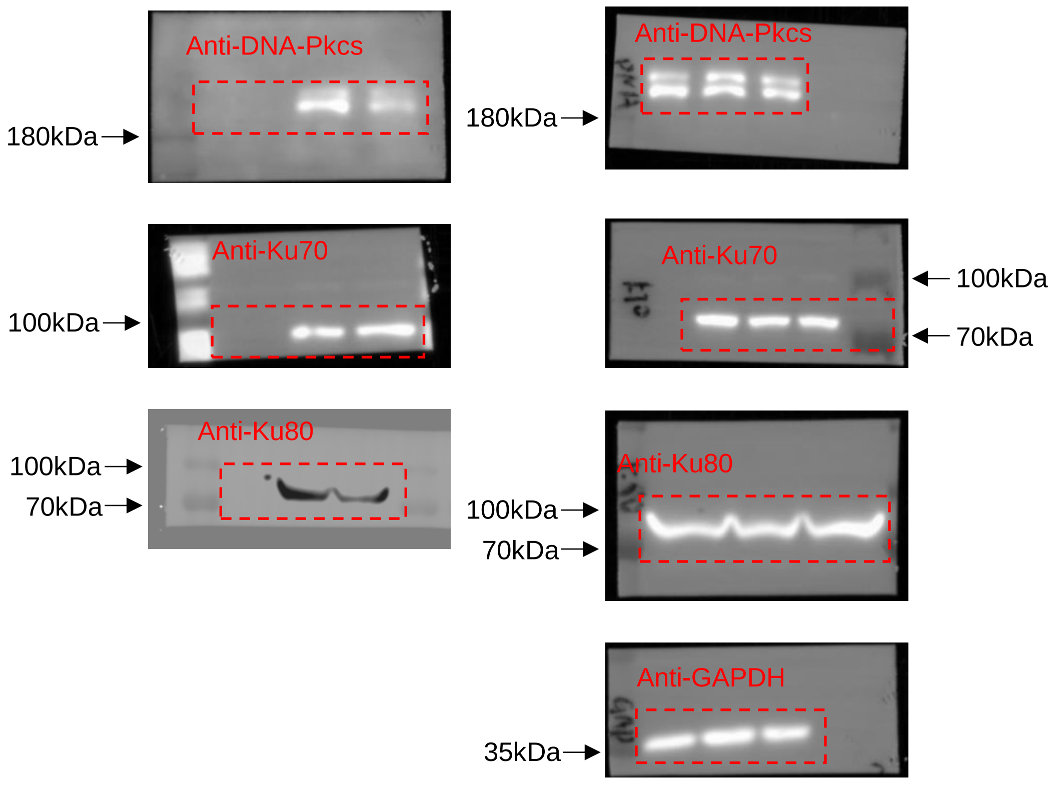

Uncropped blot of Fig.7G

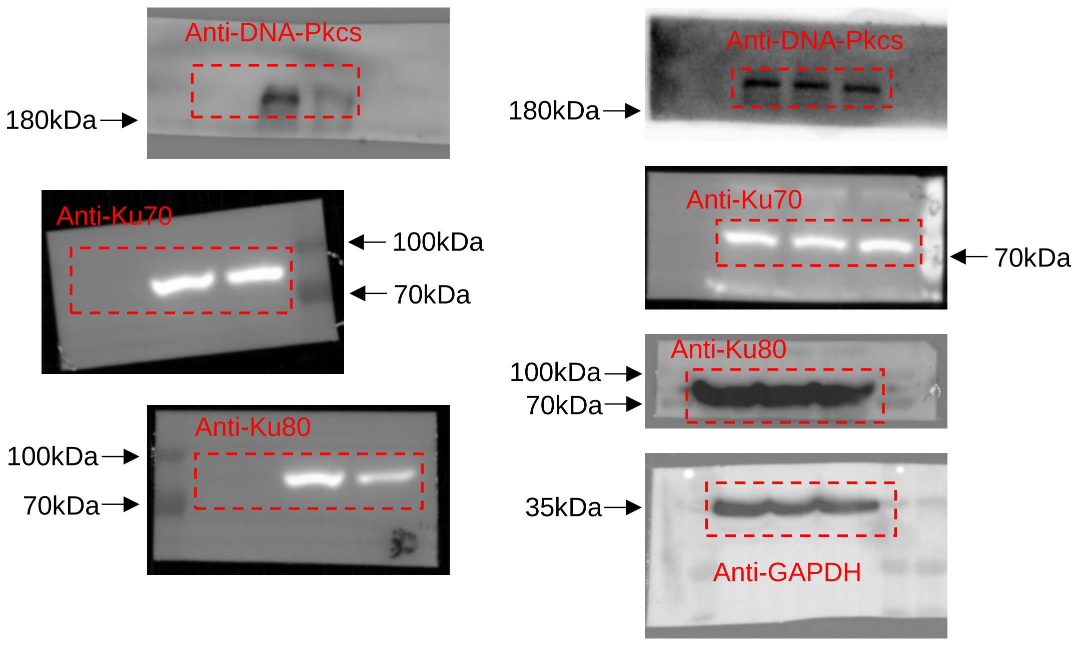

Uncropped blot of supplemental Fig.5B

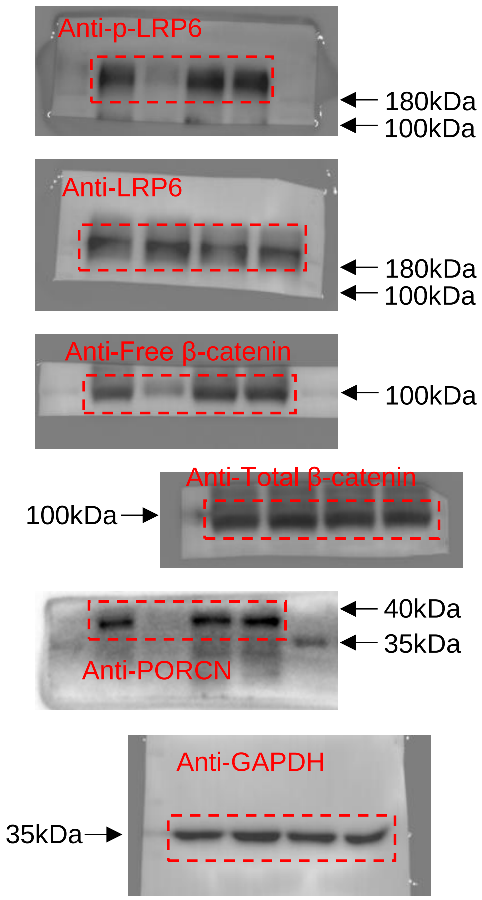

Uncropped blot of supplemental Fig.5E

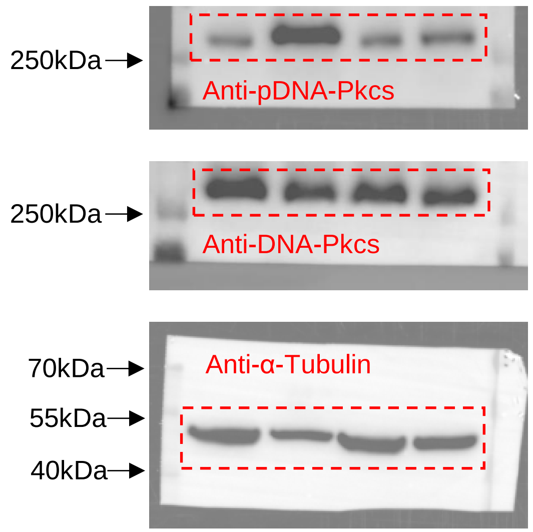

Uncropped blot of supplemental Fig.6A

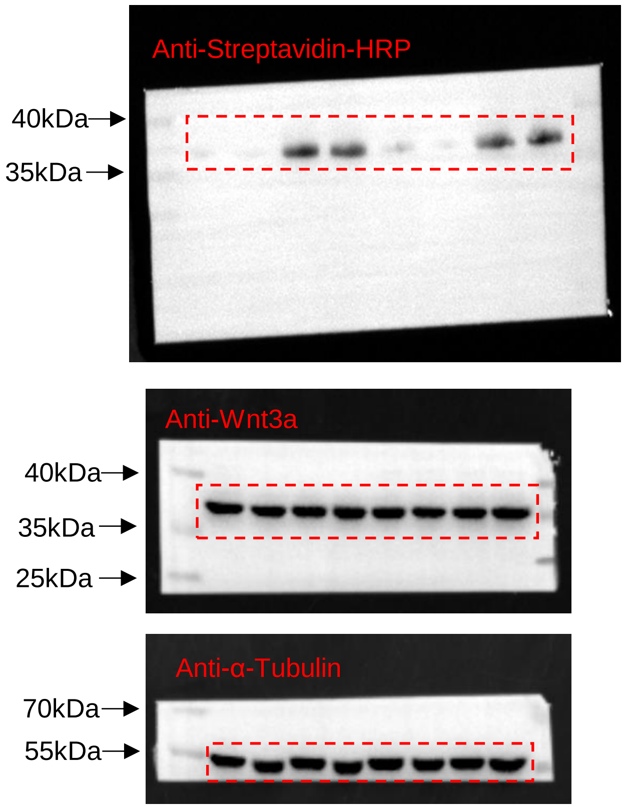

Uncropped blot of supplemental Fig.7B

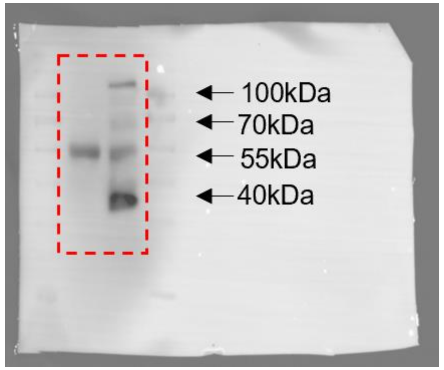

Uncropped blot of supplemental Fig.7C

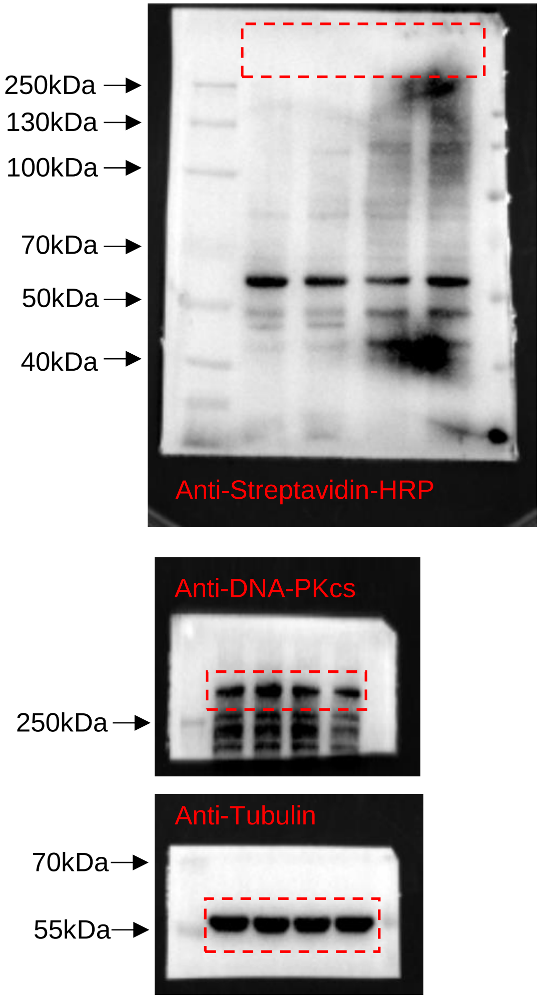

Uncropped blot of supplemental Fig.7D

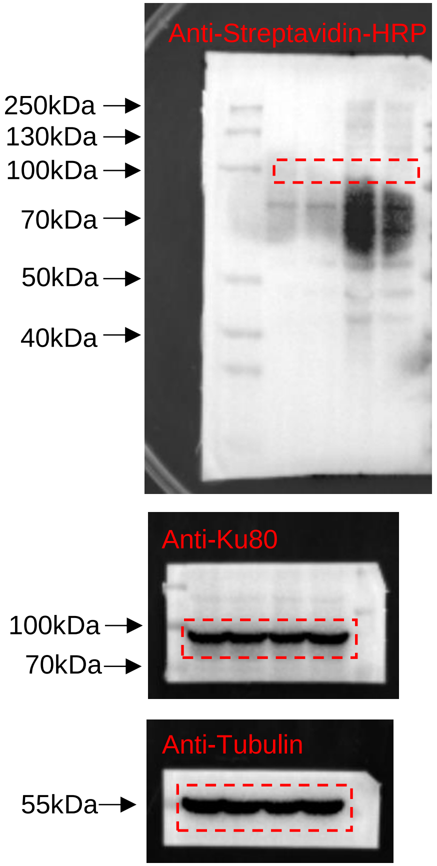

Uncropped blot of supplemental Fig.8A

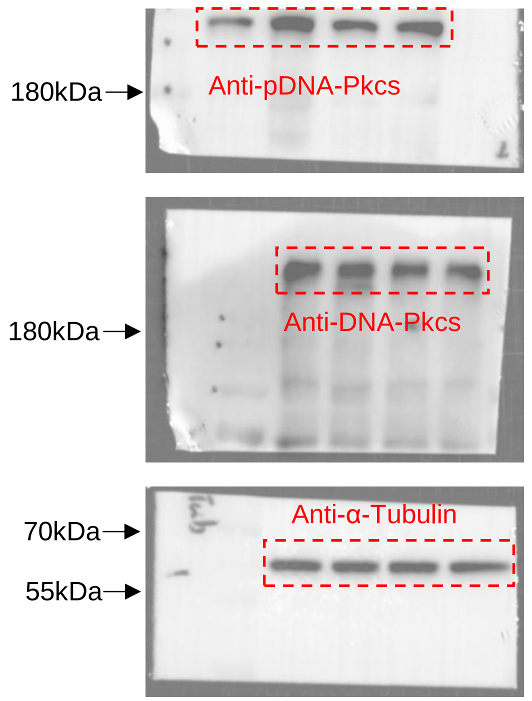

Uncropped blot of supplemental Fig.8B

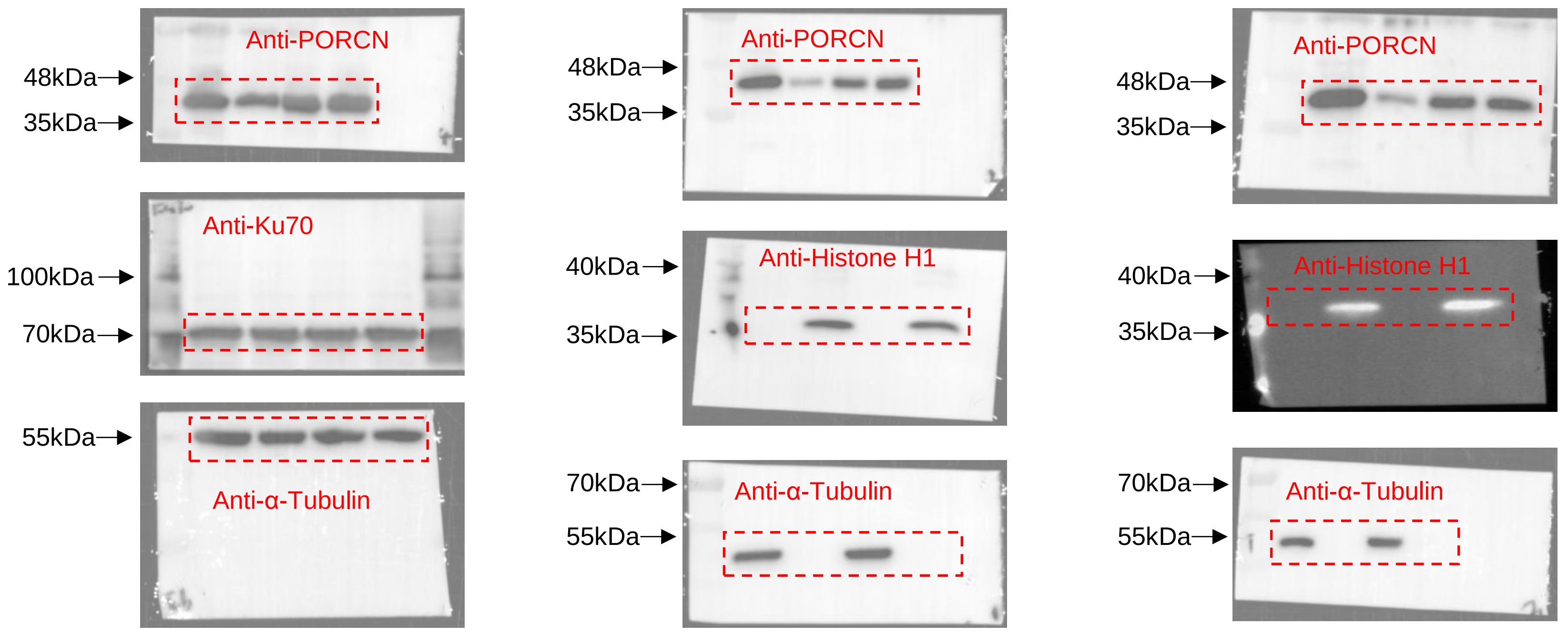

Uncropped blot of supplemental Fig.8C

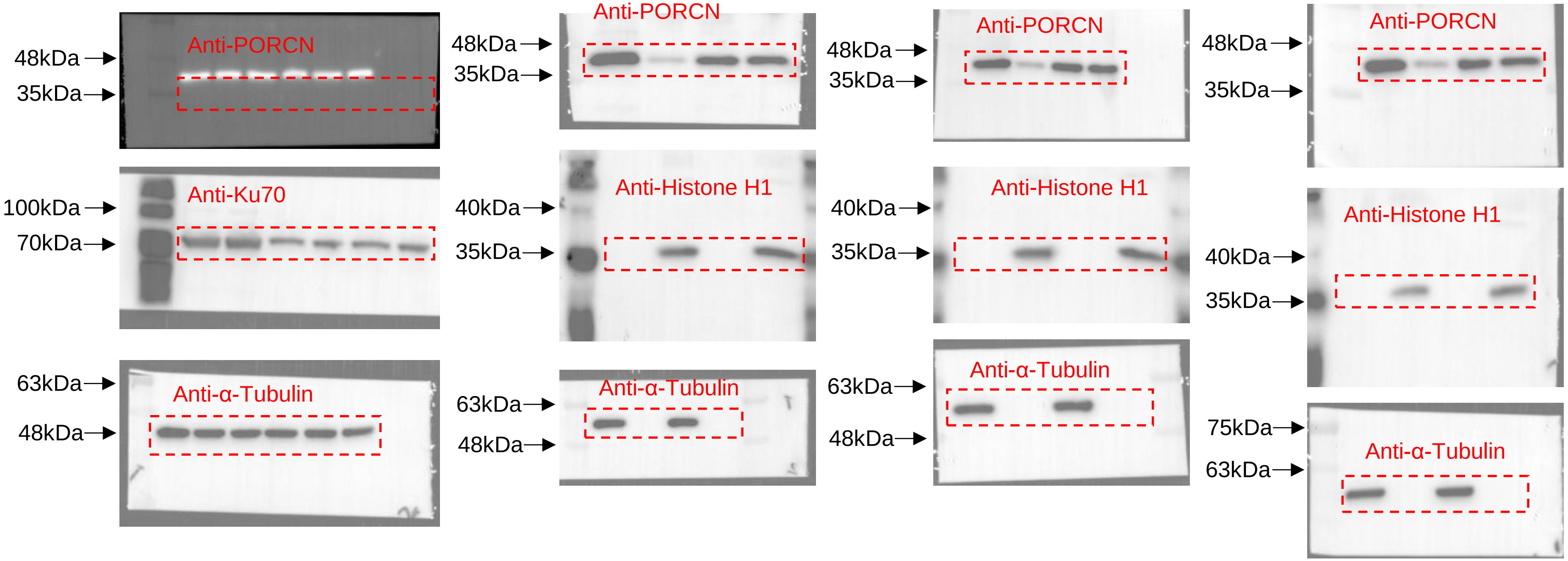

Uncropped blot of supplemental Fig.8D

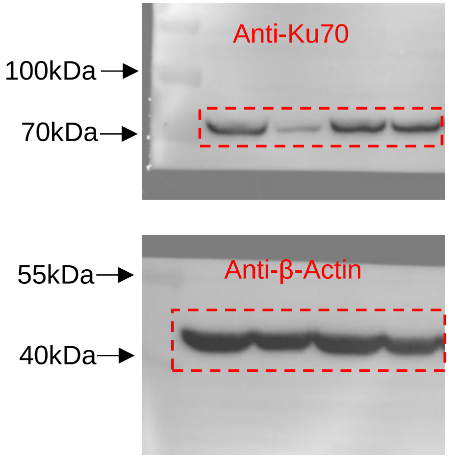

Supplement: Supplementary file 2 — Supplementary Material 2 [file 40164_2024_572_MOESM2_ESM.pdf]
